# Modeling the effects of meteorological factors and media-driven public awareness on seasonal influenza outbreaks

**DOI:** 10.1371/journal.pone.0342962

**Published:** 2026-06-08

**Authors:** Chunya Liu, Hua Liu, Yumei Wei, Jianhua Ye, Gang Ma, Weide Li, Shujuan Hu

**Affiliations:** 1 School of Mathematics and Computer Science, Northwest Minzu University, Lanzhou, Gansu, China; 2 School of Mathematics and Statistics, Lanzhou University, Lanzhou, Gansu, China; 3 Key Laboratory of Linguistic and Cultural Computing Ministry of Education, Northwest Minzu University, Lanzhou, Gansu, China; 4 Experimental Teaching Department, Northwest Minzu University, Lanzhou, Gansu, China; 5 College of Atmospheric Sciences, Lanzhou University, Lanzhou, Gansu, China; Parahyangan Catholic University: Universitas Katolik Parahyangan, INDONESIA

## Abstract

Seasonal influenza remains a significant public health challenge in China, with its transmission dynamics influenced by both environmental conditions and media-driven public awareness. In this study, we developed a compartmental model that incorporates media-induced public awareness and meteorological factors to investigate the complex transmission mechanisms of influenza across different provinces in China. We conducted theoretical analyses to characterize the model’s dynamical behavior, including conditions for disease eradication and persistence. Model parameters were estimated based on provincial data on influenza cases, Baidu search indices, and ERA5 reanalysis meteorological data through MCMC methods, providing province-specific parameters and corresponding time-varying effective reproduction numbers. Our results indicate that both low temperatures and reduced precipitation significantly facilitate influenza transmission, while media-driven public awareness plays a critical role in reducing transmission risk. A decline in media influence tends to increase the epidemic peak intensity. We observed considerable regional heterogeneity in the responses to media influence and climate variables: provinces like Zhejiang, Sichuan, Henan and Anhui are more sensitive to media influence, whereas regions like Xinjiang, Gansu, and Chongqing show limited responses. Overall, temperature was found to exert a stronger regulatory effect on influenza transmission than total precipitation. However, in northwestern provinces such as Gansu and Xinjiang, the influence of temperature on influenza transmission is relatively weaker during the winter season, while the effect of precipitation becomes more pronounced. Finally, we projected influenza trends for the first half of 2025. Forecasts show an increasing trend in influenza activity in most provinces, with model predictions closely matching observed data.

## 1 Introduction

Seasonal influenza is a widespread and highly transmissible acute respiratory illness caused by influenza viruses, primarily spread through respiratory droplets, direct contact, and contaminated surfaces. According to estimates from the World Health Organization [1], this disease affects nearly one billion individuals globally each year, with millions developing severe symptoms and several hundred thousand succumbing to respiratory complications. In 2024 alone, influenza cases in China surged to 8,588,661 [2]. The seasonal outbreaks of influenza impose substantial strain on healthcare systems, often leading to bed shortages and resource constraints, particularly during the winter and early spring months in temperate regions. Consequently, a thorough understanding of the transmission dynamics and influencing factors of influenza is critical for developing precise and effective intervention strategies to reduce the overall disease burden.

There are numerous studies indicating that meteorological factors significantly influence the transmission of influenza. Temperature, humidity, and precipitation significantly affect the survival and airborne transmission of influenza viruses, influencing their infectivity and overall disease burden. Cold and dry conditions enhance the survival of influenza viruses in the environment, thereby elevating the likelihood of transmission [3]. Chong et al. [4] analyzed data from 45 counties in Japan and found that higher absolute humidity significantly reduced the risk of influenza, particularly in lower latitude regions. Shi et al. [5] indicated that influenza activity during the Spanish flu was closely related to dew point temperature and precipitation levels. Liu et al. [6] studied influenza cases in Shaoyang, China, and found that a 5°C decrease in minimum temperature led to an 8% increase in influenza cases after a one-week delay, with the effect being modulated by relative humidity. Similarly, Jing et al. [7] examined influenza transmission in Gansu Province and found that low temperatures, low humidity, and low precipitation levels facilitated viral spread, with temperature being the most influential factor. Furthermore, Qi et al. [8] investigated influenza patterns in Chongqing and discovered that influenza activity was negatively correlated with temperature, humidity, and wind speed, where lower temperatures and humidity levels notably amplified the risk of infection.

Although many studies have established a connection between meteorological factors and influenza spread, the inclusion of this factor in transmission dynamics models is still lacking. The function $\delta (T)=\alpha e^{\gamma T}$, proposed by Handel et al. [9], models the decay of influenza viruses in the environment, where T stands for temperature, α and γ are constants. Building upon this framework, Xing et al. [10] further investigated the role of temperature-dependent decay in the recurrence of H7N9 avian influenza in China. Additionally, Jing et al. [7] employed the $\delta (T)=\alpha e^{\gamma T}$ function in their study of seasonal influenza outbreaks in Gansu Province, integrating relative humidity and total precipitation data to comprehensively assess the key influencing factors.

The impact of media-driven public awareness on disease dynamics has gained increasing attention in recent years. The media significantly influences public attitudes and actions concerning health, which can affect the transmission of infectious diseases. Information about disease outbreaks, preventive measures, and vaccinations, disseminated through news, social media, and health initiatives, can have a profound impact on public behavior. Cui et al. [11] developed a model incorporating a contact and transmission term of $\mu e^{-mI}$, which indicates that the contact transmission rate is not only related to the virus’s transmission capability but also closely linked to the level of awareness of each susceptible individual in the population. The parameter m>0 quantifies the impact of media coverage on disease dynamics. Wu et al. [12] explored the effect of media reporting on disease transmission by modifying the transmission coefficient, replacing β with $\beta e^{-a_{1}E-a_{2}I-a_{3}H}$, where $a_{1}$, $a_{2}$, and $a_{3}$ represent the psychological influence of media on different population groups. These terms reflect how media reports influence public psychology and behavior. Liu et al. [13] proposed a compartmental model incorporating the parameter $\beta_{0}e^{-bC\left(t\right)}$ to characterize how media coverage influences public psychology and behavior in response to reported cervical cancer cases, suggesting that media reporting could serve as an effective intervention. Yan et al. [14] examined the influence of individual behavioral changes (media) on the COVID-19 epidemic by embedding $e^{-kp\left(t\right)}$ into the transmission rate. Here, we do not provide a detailed discussion of each study individually but rather highlight the overall impact of media on disease dynamics. Interested readers may refer to the cited literature and their references for further details.

Although media-driven public awareness is recognized as a key factor in epidemic control, relatively few mathematical models have incorporated its impact into provincial-level analyses of influenza transmission in China. Media reports disseminated via news outlets, social media platforms, and health campaigns can rapidly convey information on influenza outbreaks, preventive measures, and vaccination, thereby significantly altering public behavior and response [15]. This media-induced shift in public awareness directly alters how often susceptible individuals come into contact with those who are infected, thereby influencing the effectiveness of disease control measures.

Public health awareness and disease prevention behaviors are often evident in the active search for health-related information. The Baidu Search Index (BSI) quantifies the frequency of specific keyword searches on the Baidu search engine and serves as a valuable proxy for public interest and awareness of various health-related issues. In recent years, BSI has garnered increasing scholarly interest and is regarded as an effective indicator of collective public awareness. By analyzing fluctuations in BSI, it is possible to quantitatively assess the public’s sensitivity and response speed to health information dissemination, thereby providing an innovative data source and analytical perspective for investigating the impact of media reporting on influenza transmission [16]. Leveraging such data not only reveals the spatiotemporal evolution of public health awareness but also facilitates the integration of media effects into influenza transmission dynamic models, ultimately enabling the construction of more precise and adaptive infectious disease models.

Motivated by the above description and the characteristics of influenza transmission, we have developed a new transmission dynamics model that integrates both media-driven public awareness and meteorological factors. The structure of this paper is outlined below: Section 2 presents the formulation of the non-autonomous transmission dynamics model. Section 3 provides a detailed analysis of the model, including its effective reproduction number, global asymptotic stability of the disease-free periodic solution, and the model’s uniform persistence; Section 4 employs MCMC algorithms to fit optimal parameters and compute the effective reproduction numbers for various provinces in China, based on influenza case data and the BSI from 2023 to 2024; Section 5 conducts a comprehensive sensitivity analysis to identify the key parameters influencing influenza transmission in different provinces; and Section 6 offers predictions regarding the future trends of influenza; Section 7 concludes with a summary and discussion.

## 2 Model formulation

In addition to the traditional SIR model, we introduce an additional compartment to represent influenza viruses in the environment. Let V(t) denote the concentration of influenza viruses in contaminated environments, including airborne aerosols and fomites. Based on the classical SIR framework, we define the transmission rate as β(t), so that the direct transmission term between susceptible and infected individuals is given by β(t)SI.

Since influenza viruses can be transmitted via both airborne aerosols and fomite contamination [17,18], the indirect transmission term is modeled as β(t)θSV, where θ quantifies the relative reduction in transmission efficiency through indirect pathways compared to direct transmission. Meteorological conditions, particularly temperature and humidity, have been shown to affect influenza virus persistence in the environment by influencing viral stability in aerosols and on surfaces. To incorporate these effects, the virus clearance rate is defined as a time-dependent function, ρ(t)=ρ(T(t), P(t)), where T(t) denotes the temperature and P(t) denotes the total precipitation at time t.

To assess the influence of media coverage and disease-related information on the transmission dynamics of influenza, we introduce an additional compartment, K(t), which represents the search index for influenza-related keywords. This index is assumed to be driven by the collective behavior of susceptible, infected, and recovered individuals [14,19]. Given the differences in risk perception and health information-seeking behaviors, we hypothesize that susceptible individuals contribute the least to the search index. This assumption is based on the rationale that, compared to infected or recovered individuals, susceptible individuals often lack symptoms and formal diagnoses, leading to lower awareness of health risks and less motivation to seek disease-related information. In contrast, infected and recovered individuals are more likely to actively search for information regarding symptoms, treatment options, or disease progression. This conclusion is consistent with the findings from numerical simulations in the literature [20]. To reflect the transmission-reducing influence of media, we introduce an attenuation factor $e^{-\delta K\left(t\right)}$ into the incidence terms. Accordingly, we refine the direct and indirect transmission terms as $\beta \left(t\right)SIe^{-\delta K\left(t\right)}$ and $\beta \left(t\right)\theta SVe^{-\delta K\left(t\right)}$, respectively.

**Fig 1:**
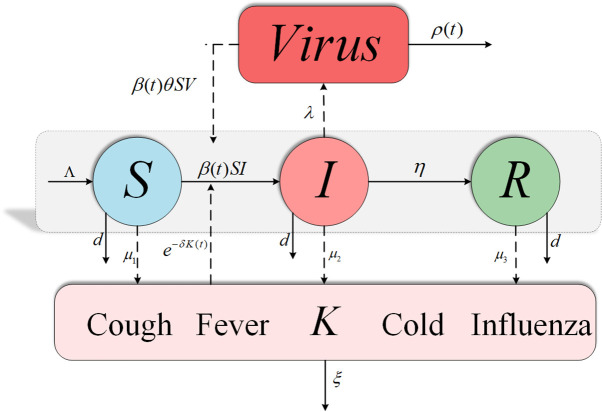
Compartment diagram of the influenza model. The “Virus” compartment represents the influenza virus, while *K* denotes media coverage and disease-related information (BSI). The terms “Cough”, “Fever”, “Cold” and “Influenza” indicate the influenza-related keywords, which are discussed in detail in Section 4.


The total number of individuals at time t is expressed as N(t)=S(t)+I(t)+R(t). All parameters and periodic functions (with period ω) are assumed to be positive (see Table 1), The flow diagram is shown in Fig 1, and the model is given below,

**Table 1:** Description of Model Parameters.

Parameter	Description
Λ	Rate of susceptible individuals entering the population (month^−1^)
d	Natural mortality rate (month^−1^)
δ	Regulatory coefficient of media-driven public awareness on influenza transmission behavior
λ	The rate at which infected individuals shed the virus during the infectious period (month^−1^)
η	The rate at which infected individuals recover (month^−1^)
$\mu_{\text{1}}$	Rate at which susceptible individuals contribute to the search index
$\mu_{\text{2}}$	Rate at which infected individuals contribute to the search index
$\mu_{\text{3}}$	Rate at which recovered individuals contribute to the search index
ξ	Natural dissipation rate of media influence (month^−1^)
θ	Indirect transmission coefficient
β(t)	The transmission rate from infected to susceptible individuals (person^−1^month^−1^)
ρ(t)	The rate at which the influenza virus is cleared

$$\begin{array}{c} \left\{ \begin{array}{c} @l@\frac{\text{d}S}{\text{d}t}=\Lambda -\beta \left(t\right)e^{-\delta K\left(t\right)}(SI+\theta SV)-dS,\mathrm{\backslash vspace}1.0mm\\ \frac{\text{d}I}{\text{d}t}=\beta \left(t\right)e^{-\delta K\left(t\right)}(SI+\theta SV)-(d+\eta )I,\mathrm{\backslash vspace}1.0mm\\ \frac{\text{d}R}{\text{d}t}=\eta I-dR,\mathrm{\backslash vspace}1.0mm\\ \frac{\text{d}V}{\text{d}t}=\lambda I-\rho \left(t\right)V,\mathrm{\backslash vspace}1.0mm\\ \frac{\text{d}K}{\text{d}t}=\mu_{\text{1}}S+\mu_{\text{2}}I+\mu_{\text{3}}R-\xi K.\mathrm{\backslash vspace}1.0mm \end{array}\right. \end{array}$$ (1)

**Theorem 2.1** Define the region


$$\begin{array}{c} \begin{array}{c} \Gamma =\backslash \{(S,\;I,\;R,\;V,\;K)\in \overset{5}{\underset{+}{ℜ}}:\;0\leq S,\;I,\;R,\;N=S+I+R\leq \frac{\Lambda }{d},\;0\leq V\leq \frac{\lambda \Lambda }{dM},\;\\ 0\leq K\leq \frac{\Lambda (\mu_{1}+\mu_{3}+\mu_{3})}{d\xi }\}.\; \end{array} \end{array}$$

Then, model (1) is bounded in Γ, which is a positively invariant set. If the initial conditions satisfy S(0)>0,I(0)>0,R(0)>0,V(0)>0 and K(0)>0, then the solution (S(t),I(t),R(t),V(t),K(t)) remains strictly positive for all t≥0.

**Proof**. Let


$$\begin{array}{c} W\left(t\right)=min\left\{S\left(t\right),\;I\left(t\right),\;R\left(t\right),V\left(t\right),K\left(t\right)\right\},\;\forall t\geq 0\text{.} \end{array}$$

Since W(0)>0. assume by contradiction that $W\left(t_{1}\right)=0$ for some $t_{1}>0$, while W(t)>0 for $t\in \left[0,\;t_{1}\right)$.

If $W\left(t_{1}\right)=S\left(t_{1}\right),$ then all other variables remain nonnegative on $t\in \left[0,\;t_{1}\right)$. Using the first equation of system (1), we derive


$$\begin{array}{c} \frac{\text{d}S}{\text{d}t}\geq -\beta \left(t\right)e^{-\delta K\left(t\right)}(I+\theta V)S-dS,\;\forall t>0\text{.} \end{array}$$

We have


$$\begin{array}{c} 0=S\left(t_{1}\right)\geq S\left(0\right)e^{-\overset{t_{1}}{\underset{0}{\int }}[\beta \left(\zeta \right)e^{-\delta K\left(\zeta \right)}\left(I+\theta V\right)+d]d\zeta }>0. \end{array}$$

This leads to a contradiction, so we conclude that S(t)>0,∀t≥0.

In the same way, it can be shown that I(t)≥0,R(t)≥0,V(t)≥0 and K(0)>0 for all t≥0.

Next, we prove the uniform and ultimate boundedness of the solution to model (1).


$$\begin{array}{c} \frac{\text{d}N}{\text{d}t}=\frac{\text{d}S}{\text{d}t}+\frac{\text{d}I}{\text{d}t}+\frac{\text{d}R}{\text{d}t}=\Lambda -d(S+I+R)=\Lambda -dN, \end{array}$$

by applying the comparison theorem, we can get $\underset{t\to \infty }{limsup}N\left(t\right)\leq \frac{\Lambda }{d}.$

Considering the fourth equation of model (1), we derive


$$\begin{array}{c} \frac{\text{d}V}{\text{d}t}=\lambda I-\rho \left(t\right)V\leq \lambda \frac{\Lambda }{d}-\rho \left(t\right)V. \end{array}$$

Given that ρ(t) is continuous, positive, and periodic, it is clear that ρ(t) is bounded. Let M=min{ρ(t),∀t≥0}, we have


$$\begin{array}{c} \frac{\text{d}V}{\text{d}t}\leq \frac{\lambda \Lambda }{d}-\rho \left(t\right)V\leq \frac{\lambda \Lambda }{d}-MV. \end{array}$$

This implies $\underset{t\to \infty }{limsup}V\left(t\right)\leq \frac{\lambda \Lambda }{dM}.$

Similarly, for the fifth equation of model (1), a similar approach can be applied to obtain $\underset{t\to \infty }{limsup}K\left(t\right)\leq \frac{\Lambda (\mu_{1}+\mu_{2}+\mu_{3})}{d\xi }.$ Thus, we derive the positive invariant set of model (1) as


$$\begin{array}{c} \begin{array}{c} \Gamma =\{(S,I,R,V,K)\in \overset{5}{\underset{+}{ℜ}}:0\leq S,I,R,\;N=S+I+R\leq \frac{\Lambda }{d},\;0\leq V\leq \frac{\lambda \Lambda }{dM},\;\\ 0\leq K\leq \frac{\Lambda (\mu_{1}+\mu_{2}+\mu_{3})}{d\xi }\}. \end{array} \end{array}$$ (2)

## 3 Model analysis

### 3.1 Effective reproduction number

In infectious disease dynamics, the effective reproduction number $R_{e}$ is a key indicator that quantifies the average number of secondary infections generated by an infectious individual under actual epidemic conditions and intervention measures. In the following section, we employ the approach proposed by Wang and Zhao [21] to calculate $R_{e}$. The disease-free periodic solution


$$\begin{array}{c} P_{0}=\left(S_{0},\;I_{0},\;R_{0},\;V_{0},\;K_{0}\right)=\left(\frac{\Lambda }{d},0,0,0,\frac{\mu_{\text{1}}\Lambda }{d\xi }\right) \end{array}$$

of model (1) can be easily computed. Let $x=(I,R,V,K,S{)}^{T}$, model (1) can be expressed as


$$\begin{array}{c} \frac{dx}{dt}=ℱ\left(t,x\right)-𝒱\left(t,x\right), \end{array}$$

where


$$\begin{array}{c} ℱ\left(t,x\right)=\left[\begin{array}{c} @c@\beta \left(t\right)e^{-\delta K\left(t\right)}(SI+\theta SV)\\ 0\\ 0\\ 0\\ 0 \end{array}\right],\text{\textbackslash{}hspace\{0.17em}\}𝒱\left(t,x\right)=\left[\begin{array}{c} @c@(d+\eta )I\\ -\eta I+dR\\ -\lambda I+\rho \left(t\right)V\\ -\mu_{\text{1}}S-\mu_{\text{2}}I-\mu_{\text{3}}R+\xi K\\ -\Lambda +\beta \left(t\right)e^{-\delta K\left(t\right)}(SI+\theta SV)+dS \end{array}\right]. \end{array}$$

The conditions (A1)–(A5) in Wang and Zhao [21] are clearly met. Let f(t,x(t))=ℱ(t,x)−𝒱(t,x), and define


$$\begin{array}{c} M\left(t\right):=\left(\frac{\partial f_{i}\left(t,x^{0}\left(t\right)\right)}{\partial x_{i}}\right),\;i=4,5, \end{array}$$ (3)

where the disease-free periodic solution is $x^{0}\left(t\right)=\left(0,0,0,\frac{\mu_{1}\Lambda }{d\xi },\frac{\Lambda }{d}\right)$. The ω -periodic system $\frac{\text{d}z}{\text{d}t}=M\left(t\right)z,$ admits a monodromy matrix, here denoted by $\Phi_{M}\left(t\right)$. It is clear that $\rho \left(\Phi_{M}\left(\omega \right)\right)=max\left\{e^{-dt},e^{-\xi t}\right\}<1,$ where $\rho \left(\Phi_{M}\left(\omega \right)\right)$ denotes the spectral radius of $\Phi_{M}\left(\omega \right)$. Clearly, the condition (A6) proposed by Wang and Zhao [21] is also fulfilled.

Next, we verify condition (A7) by defining


$$\begin{array}{c} F\left(t\right)={\left(\frac{\partial {ℱ}_{i}\left(t,x^{\text{0}}\left(t\right)\right)}{\partial x_{j}}\right)}_{1\leq i,j\leq 3},\text{\textbackslash{}hspace\{0.17em}\}V\left(t\right)={\left(\frac{\partial {𝒱}_{i}\left(t,x^{\text{0}}\left(t\right)\right)}{\partial x_{j}}\right)}_{1\leq i,j\leq 3} \end{array}$$ (4)

The i-th components of ℱ(t,x) and 𝒱(t,x) are denoted as ${ℱ}_{i}\left(t,x\right)$ and ${𝒱}_{i}\left(t,x\right)$, respectively. Thus, we obtain


$$\begin{array}{c} F\left(t\right)=\left[\begin{array}{ccc} @ccc@\frac{\Lambda \beta \left(t\right)}{d}e^{-\delta \left(\frac{\mu_{\text{1}}\Lambda }{d\xi }\right)} & 0 & \frac{\Lambda \theta \beta \left(t\right)}{d}e^{-\delta \left(\frac{\mu_{\text{1}}\Lambda }{d\xi }\right)}\\ 0 & 0 & 0\\ 0 & 0 & 0 \end{array}\right],\; \end{array}$$ (5)

and


$$\begin{array}{c} V\left(t\right)=\left[\begin{array}{ccc} @ccc@d+\eta & 0 & 0\\ -\eta & d & 0\\ -\lambda & 0 & \rho \left(t\right) \end{array}\right]. \end{array}$$ (6)

Clearly, F(t) has only non‑negative entries, and the fact that every off‑diagonal element of −V(t) is likewise non‑negative means −V(t) is cooperative.

Furthermore, let Y(t,s), for t≥s, represent the evolution operator associated with the linear ω-periodic model,


$$\begin{array}{c} \frac{dy}{dt}=-V\left(t\right)y, \end{array}$$ (7)

for each s∈ℜ, the 3 × 3 matrix Y(t,s) satisfies the following conditions.


$$\begin{array}{c} \text{d}Y\left(t,s\right)/\text{d}t=-V\left(t\right)Y\left(t,s\right),\;\forall t\geq s, \end{array}$$ (8)

Let Y(s,s)=I denote the 3 × 3 identity matrix. The monodromy matrix of the linear of model dY(t,s)/dt=−V(t)Y(t,s) is given by $\Phi_{-V}\left(t\right)$. Since $\rho \left(\Phi_{-V}\left(\omega \right)\right)<1,$ it follows that condition (A7) is satisfied.

Following the framework introduced by Wang and Zhao [21], ϕ(s) is taken to be ω-periodic, representing the initial distribution of infected individuals. Thus, F(s)ϕ(s) represents the distribution of new infections caused by individuals at time s. Y(t,s)F(s)ϕ(s) refers to those who were newly infected by ϕ(s) and remain in the infected state at time s for t≥s. Let


$$\begin{array}{c} \psi \left(t\right):=\int_{-\infty }^{t}Y\left(t,s\right)F\left(s\right)\phi \left(s\right)ds=\int_{\text{0}}^{\infty }Y(t,t-a)F(t-a)\phi (t-a)da,\;a\in [0,\infty ), \end{array}$$ (9)

where ψ(t) represents the cumulative count of new cases caused by previously infected individuals, as characterized by ϕ(s), up to time t.

Consider the Banach space $C_{\omega }$, consisting of all real-valued ω-periodic functions from ℜ to ${ℜ}^{3}$, equipped with the supremum norm ||·||. Define the positive cone $\overset{+}{\underset{\omega }{C}}$ as the set of functions $\phi \in C_{\omega }$ satisfying ϕ(t)≥0 for all t∈ℜ. A corresponding linear operator $L:\;C_{\omega }\to C_{\omega }$ is defined as follows:


$$\begin{array}{c} \left(L\phi \right)\left(t\right)=\int_{\text{0}}^{\infty }Y(t,t-a)F(t-a)\phi (t-a)da, \end{array}$$ (10)

where ρ(L) is the spectral radius of L. Consequently, the effective reproduction number for the periodic epidemic model (1) is given by


$$\begin{array}{c} R_{e}:=\rho \left(L\right), \end{array}$$ (11)

**Lemma 2.1** [Theorem 2.2 [21]] The following statements hold:

(1) $R_{e}=1$ if and only if $\rho \left(\Phi_{F-V}\left(\omega \right)\right)=1.$
(2) $R_{e}>1$ if and only if $\rho \left(\Phi_{F-V}\left(\omega \right)\right)>1.$
(3) $R_{e}<1$ if and only if $\rho \left(\Phi_{F-V}\left(\omega \right)\right)<1.$

Therefore, the periodic solution $x^{0}\left(t\right)$ corresponding to the absence of disease, remains locally asymptotically stable when $R_{e}<1$, whereas it becomes unstable if $R_{e}>1$.

To determine $R_{e}$ for the periodic epidemic model (1), we adopt the methodology developed by Wang and Zhao [21], examine the following linear system with period ω,


$$\begin{array}{c} \frac{d\omega }{dt}=\left[\frac{F\left(t\right)}{\lambda_{1}}-V\left(t\right)\right]\omega ,\;t\in ℜ,\;\lambda_{1}\in \left(0,\infty \right), \end{array}$$ (12)

where the coefficient matrix is defined as


$$\begin{array}{c} \frac{F\left(t\right)}{\lambda_{\text{1}}}-V\left(t\right)=\left[\begin{array}{ccc} @ccc@\frac{\Lambda \beta \left(t\right)}{\lambda_{\text{1}}d}e^{-\delta \left(\frac{\mu_{\text{1}}\Lambda }{\xi d}\right)}-(d+\eta ) & 0 & \frac{\Lambda \theta \beta \left(t\right)}{\lambda_{\text{1}}d}e^{-\delta \left(\frac{\mu_{\text{1}}\Lambda }{\xi d}\right)}\\ \eta & -d & 0\\ \lambda & 0 & -\rho \left(t\right) \end{array}\right]. \end{array}$$ (13)

Let $W\left(t,\;s,\;\lambda_{1}\right)$ denote the evolution operator associated with system (13) in ${ℜ}^{3}$ defined for t>s with s∈ℜ. Based on this operator, we implement a numerical algorithm to determine the effective reproduction number.

**Lemma 2.2** [Theorem 2.1 [21]] The following statements hold

(1) If $\rho \left(W\left(\omega ,\;0,\;\lambda_{1}\right)\right)=1$ has a positive solution $\lambda_{0},$ then $\lambda_{0}$ is an eigenvalue of L, and hence $R_{e}>0.$
(2) If $R_{e}>0$, then $\lambda_{1}=R_{e}$ is the unique solution of $\rho \left(W\left(\omega ,\;0,\;\lambda_{1}\right)\right)=1.$
(3) $R_{e}=0$ if and only if $\rho \left(W\left(\omega ,\;0,\;\lambda_{\text{1}}\right)\right)<1$ for all $\lambda_{1}>0.$

### 3.2 Extinction of the disease

**Theorem 3.1** If $R_{e}<1$, The disease-free periodic solution $x^{0}\left(t\right)$ of system (1) is globally asymptotically stable within the positively invariant set Γ.

**Proof.** By Lemma 2.1, when $R_{e}<1,$ it is sufficient to prove that $x^{0}\left(t\right)=\left(0,0,0,\frac{\mu_{\text{1}}\Lambda }{\xi d},\frac{\Lambda }{d}\right)$ is globally attractive.

From model (1) and Theorem 2.1, we obtain


$$\begin{array}{c} \underset{t\to \infty }{limsup}S\left(t\right)\leq \frac{\Lambda }{d},\text{\textbackslash{}hspace\{0.17em}\}\underset{t\to \infty }{limsup}K\left(t\right)\leq \frac{\Lambda (\mu_{\text{1}}+\mu_{\text{2}}+\mu_{\text{3}})}{d\xi }. \end{array}$$

Therefore, ∀κ>0, $\exists \;\overset{―}{t}>0$ such that


$$\begin{array}{c} S\left(t\right)\leq \frac{\Lambda }{d}+\kappa ,\text{\textbackslash{}hspace\{0.17em}\}K\left(t\right)\leq \frac{\Lambda (\mu_{\text{1}}+\mu_{\text{2}}+\mu_{\text{3}})}{d\xi }+\kappa ,\text{\textbackslash{}hspace\{0.17em}\}for\text{\textbackslash{}hspace\{0.17em}\}t>\overset{―}{t}. \end{array}$$

We next examine the comparison system.


$$\begin{array}{c} \left\{ \begin{array}{c} @l@\frac{\text{d}\hat{I}}{\text{d}t}=\beta \left(t\right)e^{-\delta \left(\frac{\Lambda (\mu_{\text{1}}+\mu_{\text{2}}+\mu_{\text{3}})}{d\xi }+\kappa \right)}(\hat{I}+\theta \hat{V})(\frac{\Lambda }{d}+\kappa )-(d+\eta )\hat{I},\\ \frac{\text{d}\hat{R}}{\text{d}t}=\eta \hat{I}-d\hat{R},\\ \frac{\text{d}\hat{V}}{\text{d}t}=\lambda \hat{I}-\rho \left(t\right)\hat{V}. \end{array}\right. \end{array}$$ (14)

Let $x\left(t\right)=(\hat{I}\left(t\right),\;\hat{R}\left(t\right),\;\hat{V}\left(t\right){)}^{T},$ system (14) is expressed as,


$$\begin{array}{c} \frac{\text{d}x\left(t\right)}{\text{d}t}=\Delta \left(t\right)x\left(t\right), \end{array}$$

where


$$\begin{array}{c} \Delta \left(t\right)=e^{-\delta \left(T+\kappa \right)}F\left(t\right)-V\left(t\right)+\kappa e^{-\delta \left(T+\kappa \right)}h\left(t\right),\text{\textbackslash{}hspace\{0.17em}\}T=\frac{\Lambda (\mu_{\text{2}}+\mu_{\text{3}})}{d\xi }, \end{array}$$

and


$$\begin{array}{c} h\left(t\right)=\left[\begin{array}{ccc} @ccc@\beta \left(t\right)e^{-\delta \left(\frac{\mu_{\text{1}}\Lambda }{\xi d}\right)} & 0 & \beta \left(t\right)\theta e^{-\delta \left(\frac{\mu_{\text{1}}\Lambda }{\xi d}\right)}\\ 0 & 0 & 0\\ 0 & 0 & 0 \end{array}\right]. \end{array}$$

Based on Lemma 2.1 in Zhang and Zhao [21], model (14) admits a positive *ω*-periodic solution $v\left(t\right)=e^{pt}\overset{―}{v}\left(t\right)$, where $p=\frac{\text{1}}{\omega }ln\rho \left(\Phi_{\Delta }\left(\omega \right)\right)$. Since $\rho \left(\Phi_{\Delta }\left(\omega \right)\right)$ is continuous with respect to κ and T for small values, a sufficiently small κ and T can be chosen to satisfy $\rho \left(\Phi_{\Delta }\left(\omega \right)\right)<1.$ Thus, v(t) approaches 0 as t→∞.

For any nonnegative initial values $\left(\hat{I}\left(0\right),\hat{R}\left(0\right),\hat{V}\left(0\right)\right)$ for model (14), choosing H>0 sufficiently large ensures that


$$\begin{array}{c} (\hat{I}\left(0\right),\hat{R}\left(0\right),\hat{V}\left(0\right){)}^{T}\leq H\overset{―}{v}(0). \end{array}$$

Then


$$\begin{array}{c} (\hat{I}\left(t\right),\hat{R}\left(t\right),\hat{V}\left(t\right){)}^{T}\leq Hv(t),\text{\textbackslash{}hspace\{0.17em}\}\forall t>0, \end{array}$$

where Hv(t) is also a solution of model (14). Based on the comparison principle, we conclude that


$$\begin{array}{c} (I\left(t\right),R\left(t\right),V\left(t\right){)}^{T}\leq (\hat{I}\left(t\right),\hat{R}\left(t\right),\hat{V}\left(t\right){)}^{T}\leq Hv\left(t\right),\text{\textbackslash{}hspace\{0.17em}\}\forall t>0. \end{array}$$

Thus


$$\begin{array}{c} \underset{t\to \infty }{lim}I\left(t\right)=0,\;\underset{t\to \infty }{lim}R\left(t\right)=0\text{, }\underset{t\to \infty }{lim}V\left(t\right)=0. \end{array}$$

By applying the theory of asymptotically periodic semi-flows (Theorem 3.2.1 [22]), we obtain that


$$\begin{array}{c} \underset{t\to \infty }{lim}(I\left(t\right),R\left(t\right),V\left(t\right),K\left(t\right)-\frac{\mu_{\text{1}}\Lambda }{d\xi },S(t)-\frac{\Lambda }{d})=\left(0,0,0,0,0\right). \end{array}$$ (15)

This concludes the proof.

### 3.3 Uniform persistence of the disease

**Theorem 3.2** If $R_{e}>1$, then model (1) admits at least one positive periodic solution. Furthermore, for any initial condition $x_{\text{0}}=\left(S\left(0\right),\;I\left(0\right),\;R\left(0\right),\;V\left(0\right),\;K\left(0\right)\right)$, there exists a constant χ>0 such that the solution (S(t), I(t), R(t), V(t),K(t)) satisfies


$$\begin{array}{c} \underset{t\to \infty }{liminf}\left(S\left(t\right),\;I\left(t\right),\;R\left(t\right),\;V\left(t\right),\;K\left(t\right)\right)\geq \left(\chi ,\;\chi ,\;\chi ,\;\chi ,\;\chi \right). \end{array}$$

**Proof.** Define


$$\begin{array}{c} \begin{array}{c} X:=\overset{5}{\underset{+}{ℜ}},\\ X_{\text{0}}:=\left\{(S,I,R,V,K)\in X:I>0,R>0,V>0\right\},\\ \partial X_{0}:=X\backslash X_{0}. \end{array} \end{array}$$

Let P:X→X be the Poincaré map of model (1), satisfying


$$\begin{array}{c} P\left(x_{\text{0}}\right)=\varphi \left(\omega ,x_{\text{0}}\right),\text{\textbackslash{}hspace\{0.17em}\}\forall x_{\text{0}}\in X, \end{array}$$

where ω is the period, and $\varphi \left(\omega ,\;x_{\text{0}}\right)$ represents the unique solution of model (1) with the initial condition $\varphi \left(0,\;x_{0}\right)=x_{0}.$ For any m≥0, it follows that


$$\begin{array}{c} P^{m}\left(x_{0}\right)=\varphi \left(m\omega ,\;x_{0}\right). \end{array}$$

According to Theorem 2.1, the solution of model (1) remains uniformly bounded, which shows that P is point dissipative in X. By Definition 1.1.2 and Theorem 1.1.3 in Zhao [22], P is compact and admits a global attractor.

We let


$$\begin{array}{c} M_{\partial }=\{x_{0}\in \partial X_{0}:\;P^{m}(x_{0})\in \partial X_{0},\;\forall m\geq 0\}, \end{array}$$

$$\begin{array}{c} \Gamma =\left\{\left(S,I,R,V,K\right)\in X:\;I=0,\;R=0,\;V=0\right\}. \end{array}$$

Next, we demonstrate that $M_{\partial }=\left\{(S,0,0,0,K):\;S\geq 0,\;K\geq 0\right\}$. Evidently, for any S≥0 and K≥0, the point (S,0,0,0,K) lies in $M_{\partial }$. Suppose that, for the initial condition $\left(S,I,R,V,K\right)=M_{\partial }$, at least one among I, R or V is positive. Then, the following inequality holds,


$$\begin{array}{c} I\left(t\right)\geq e^{(Q-d-\eta )t}\times \left[I\left(0\right)+\int_{0}^{t}\theta QV\left(\tau \right)e^{-(Q-d-\eta )\tau }d\tau \right]>0, \end{array}$$

$$\begin{array}{c} R\left(t\right)\geq e^{-dt}\left(R\left(0\right)+\int_{0}^{t}\eta I\left(\tau \right)e^{d\tau }d\tau \right)>0, \end{array}$$

$$\begin{array}{c} V\left(t\right)\geq e^{-Mt}\left(V\left(0\right)+\int_{0}^{t}\lambda I\left(\tau \right)e^{M\tau }d\tau \right)>0, \end{array}$$

where L=min{β(t),∀t≥0}, $Q=(\frac{\Lambda }{d}+\kappa )Le^{-\delta \left(\Lambda (\mu_{1}+\mu_{2}+\mu_{3})/d\xi +\kappa \right)},$M=min{ρ(t),∀t≥0}. For sufficiently small t>0, $\left(S\left(t\right),I\left(t\right),R\left(t\right),V\left(t\right),K\left(t\right)\right)\notin \partial X_{0}.$ Thus, we conclude that (S(t),I(t),R(t),V(t),$K\left(t\right))\notin M_{\partial }.$ This implies that $x_{0}\notin \partial X_{0}$ and $x_{0}\notin M_{\partial }.$ Consequently, as previously demonstrated, we obtain $M_{\partial }=\left\{(S,0,0,0,K):S\geq 0,K\geq 0\right\}$.

According to Theorem 3.1, the point $x^{0}=\left(\frac{\Lambda }{d},0,0,0,\frac{\mu_{1}\Lambda }{\xi d}\right)$ is globally asymptotically stable in $M_{\partial }$. Consequently, $A_{\partial }=\left\{\left(\frac{\Lambda }{d},0,0,0,\frac{\mu_{1}\Lambda }{d\xi }\right)\right\}$ serves as the maximal compact invariant set of P within $\partial X_{\text{0}}.$ It is clear that $A_{\partial }$ is both acyclic and isolated within $\partial X_{0}.$

We aim to establish that $W^{s}\left(A_{\partial }\right)\cap X_{0}=\emptyset$ and that $A_{\partial }$ is isolated in X. Since solutions depend continuously on initial conditions, a constant ℓ>0 can be found such that for any $x_{0}\in X_{0}$ satisfying $∥x_{0}-x^{0}∥\leq \ell$, the following inequality holds:


$$\begin{array}{c} \left|\varphi \left(t,x_{\text{0}})-\varphi (t,x^{\text{0}}\right)\right||\leq \varepsilon ,\text{\textbackslash{}hspace\{1em}\}\forall \varepsilon >0,\text{\textbackslash{}hspace\{1em}\}\forall t\in [0,\omega ]. \end{array}$$

holds.

Furthermore, we assert that


$$\begin{array}{c} \underset{m\to \infty }{limsup}\;d\left(P^{m}\text{(}x_{0}\text{),}x^{0}\right)\geq \ell , \end{array}$$

where d(x,y) denotes the distance between x and y. Using a contradiction argument, we assume the following inequality holds:


$$\begin{array}{c} \underset{m\to \infty }{limsup}\;d\left(P^{m}\text{(}x_{0}\text{),}x^{0}\right)<\ell ,\text{ for\textbackslash{} some }x_{0}\in X_{0}. \end{array}$$

Assuming no loss of generality, we take that the distance $d\left(P^{m}(x_{0}),\;x^{0}\right)$ remains below ℓ for all m≥0. It follows that


$$\begin{array}{c} ∥\varphi (t,\;P^{m}\left(x_{0}\right)-\varphi \left(t,\;x^{0}\right)∥<\varepsilon ,\;\forall m\geq 0,\;\forall t\in \left[0,\omega \right]. \end{array}$$ (16)

For $\begin{array}{c} \forall t\geq 0,\text{ let }t=t^{\prime }+m\omega ,\text{ where }t^{\prime }\in \left[0,\omega \right), \end{array}$ and m=[t/ω]. We then obtain


$$\begin{array}{c} ||\varphi \left(t,x_{0}\right)-\varphi \left(t,x^{0}\right)||=||\varphi \left(t^{\prime },P^{m}\left(x_{0}\right)\right)-\varphi \left(t^{\prime },x^{0}\right)||<\varepsilon ,\;\forall t\geq 0. \end{array}$$

This implies the exists a $\omega^{\prime }>0$ such that


$$\begin{array}{cc} & \frac{\Lambda }{d}-\varepsilon \leq S\left(t\right)\leq \frac{\Lambda }{d}+\varepsilon ,\text{\textbackslash{}hspace\{0.17em}\}0\leq I\left(t\right)\leq \varepsilon ,\text{\textbackslash{}hspace\{0.17em}\}0\leq R\left(t\right)\leq \varepsilon ,\text{\textbackslash{}hspace\{0.17em}\}0\leq V\left(t\right)\leq \varepsilon ,\\ & \text{\textbackslash{}hspace\{0.17em}\}\frac{\mu_{\text{1}}\Lambda }{d\xi }-\varepsilon \leq K\left(t\right)\leq \frac{\mu_{\text{1}}\Lambda }{d\xi }+\varepsilon ,\text{\textbackslash{}hspace\{0.17em}\}\frac{\Lambda }{d}-\varepsilon \leq N\left(t\right)\leq \frac{\Lambda }{d}+3\varepsilon ,\text{\textbackslash{}hspace\{0.17em}\}fort>\omega^{\prime }. \end{array}$$

We also get


$$\begin{array}{c} \frac{dI}{dt}\geq \beta \left(t\right)e^{-\delta \left(\frac{\mu_{\text{1}}\Lambda }{d\xi }-\varepsilon \right)}(I+\theta V)(\frac{\Lambda }{d}-\varepsilon )-(d+\eta )I. \end{array}$$

Consider the comparison system given by,


$$\begin{array}{c} \left\{ \begin{array}{c} @l@\frac{\text{d}\hat{I}}{\text{d}t}=\beta \left(t\right)e^{-\delta \left(\frac{\mu_{\text{1}}\Lambda }{d\xi }-\varepsilon \right)}(\hat{I}+\theta \hat{V})(\frac{\Lambda }{d}-\varepsilon )-(d+\eta )\hat{I},\\ \frac{\text{d}\hat{R}}{\text{d}t}=\eta \hat{I}-d\hat{R},\\ \frac{\text{d}\hat{V}}{\text{d}t}=\lambda \hat{I}-\rho \left(t\right)\hat{V}. \end{array}\right. \end{array}$$ (17)

Model (17) can be written in the following form,


$$\begin{array}{c} \frac{dx\left(t\right)}{dt}=\Delta_{1}\left(t\right)x\left(t\right), \end{array}$$

where


$$\begin{array}{c} \Delta_{\text{1}}\left(t\right)=e^{\delta \varepsilon }F\left(t\right)-V\left(t\right)-\varepsilon e^{\delta \varepsilon }h\left(t\right), \end{array}$$

and


$$\begin{array}{c} h\left(t\right)=\left[\begin{array}{ccc} @ccc@\beta \left(t\right)e^{-\delta \left(\frac{\mu_{\text{1}}\Lambda }{d\xi }\right)} & 0 & \beta \left(t\right)\theta e^{-\delta \left(\frac{\mu_{\text{1}}\Lambda }{d\xi }\right)}\\ 0 & 0 & 0\\ 0 & 0 & 0 \end{array}\right]. \end{array}$$

By Lemma 2.1 (Zhang and Zhao [23]), a positive ω -periodic function $\overset{⏜}{v}\left(t\right)$ satisfying $\tilde{v}\left(t\right)=e^{\tilde{p}t}\overset{\mathrm{\backslash lower}0.5em\mathrm{\backslash smash}⌢\$}{\}}v\left(t\right)$ which solves model (17). Here, $\tilde{p}=\frac{\text{1}}{\omega }ln\rho \left(\Phi \Delta 1(\omega \right))$. Note that $\rho \left(\Phi \Delta_{1}(\omega \right))>1$, ensuring $\tilde{p}$ is positive. By the comparison principle,


$$\begin{array}{c} \underset{t\to \infty }{lim}\left(I\left(t\right),R\left(t\right),V\left(t\right)\right)=(\infty ,\infty ,\infty ). \end{array}$$

This implies unbounded growth as t→∞, which contradicts 0≤I(t)≤ε, 0≤R(t)≤ε,0≤V(t)≤ε. Consequently, $W^{s}\left(A_{\partial }\right)\cap X_{0}=\emptyset$ hold, proving that $A_{\partial }$ is isolated in X. Form Theorem 1.3.1 [22], P is uniformly persistent relative to $(X_{0},\partial X_{0}).$ Theorem 3.1.1 [22] further ensures the uniform persistence of solutions to model (1) under the same conditions. Specifically, there exists ε such that for any initial condition $x_{0}=\left(S\left(0\right),I\left(0\right),R\left(0\right),V\left(0\right),K\left(0\right)\right)$ of model (1), the solution satisfies (S(t),I(t),R(t),V(t),K(t)) for model (1) satisfies


$$\begin{array}{c} \underset{t\to \infty }{liminf}\left(I\left(t\right),R\left(t\right),V\left(t\right)\right)>\left(\varepsilon ,\varepsilon ,\varepsilon \right). \end{array}$$

We demonstrate that a positive ω-periodic solution exists for model (1), which implies that P possesses a fixed point. Let $\left(S^{*}\left(0\right),I^{*}\left(0\right),R^{*}\left(0\right),V^{*}\left(0\right),K^{*}\left(0\right)\right)\in X_{\text{0}},$ where $S^{*}\left(0\right)\geq 0,$$I^{*}\left(0\right)\geq 0,$$R^{*}\left(0\right)\geq 0,$ $V^{*}\left(0\right)\geq 0$ and $K^{*}\left(0\right)\geq 0.$ To verify $S^{*}\left(0\right)>0$, suppose for contradiction that $S^{*}\left(0\right)=0$. Under this assumption, the first equation in model (1) can be expressed as:


$$\begin{array}{c} \frac{dS}{dt}=\Lambda -\beta \left(t\right)e^{-\delta K\left(t\right)}(SI+\theta SV)-dS=\Lambda -\left(\psi \left(t\right)+d\right)S, \end{array}$$

where $\psi \left(t\right)=\beta \left(t\right)e^{-\delta K\left(t\right)}(I+\theta V)$ we have


$$\begin{array}{c} \begin{array}{c} S^{*}\left(t\right)=e^{\int_{\text{0}}^{t}-(\psi (\zeta_{\text{1}})+d)\text{d}\zeta_{\text{1}}}\left[S^{*}\left(0\right)+\int_{\text{0}}^{t}\Lambda e^{\int_{\text{0}}^{\zeta_{\text{2}}}(\psi (\zeta_{\text{1}})+d)\text{d}\zeta_{\text{1}}}\text{d}\zeta_{\text{2}}\right]\mathrm{\backslash hfill}\\ \;\;\;=e^{\int_{\text{0}}^{t}-(\psi (\zeta_{\text{1}})+d)\text{d}\zeta_{\text{1}}}\int_{\text{0}}^{t}\Lambda e^{\int_{\text{0}}^{\zeta_{\text{2}}}\left(\psi \left(\zeta_{\text{1}}\right)+d\right)\text{d}\zeta_{\text{1}}}\text{d}\zeta_{\text{2}},\;\forall t\geq 0.\mathrm{\backslash hfill} \end{array} \end{array}$$

The following inequality can be derived.


$$\begin{array}{c} S^{*}\left(m\omega \right)=e^{\int_{\text{0}}^{m\omega }-(\psi (\zeta_{\text{1}})+d)d\zeta_{\text{1}}}\int_{\text{0}}^{m\omega }\Lambda e^{\int_{\text{0}}^{\zeta_{\text{2}}}(\psi \left(\zeta_{\text{1}}\right)+d)d\zeta_{\text{1}}}d\zeta_{\text{2}}>0. \end{array}$$ (21)

The periodicity of $S^{*}\left(t\right)$ implies $S^{*}\left(0\right)=S^{*}\left(m\omega \right)=0,$ for any positive integer m. However, this contradicts the established result $S^{*}\left(m\omega \right)>0$. Consequently, we conclude $S^{*}\left(0\right)>0.$ Similarly, it can be shown that $K^{*}\left(0\right)\geq 0.$

From the previous proof and the positive invariance of $X_{0}$, we deduce that


$$\begin{array}{c} \varphi \left(t,\left(S^{*}\left(0\right),I^{*}\left(0\right),R^{*}\left(0\right),V^{*}\left(0\right),K^{*}\left(0\right)\right)\right)\in \text{Int(}\overset{\text{5}}{\underset{+}{ℜ}}\text{),}\forall t>0. \end{array}$$

This demonstrates that $\left(S^{*}\left(0\right),\;I^{*}\left(0\right),\;R^{*}\left(0\right),\;V^{*}\left(0\right),\;K^{*}\left(0\right)\right)$ constitutes a positive fixed point for the mapping P. Moreover, the solution $\left(S^{*}\left(t\right),\;I^{*}\left(t\right),\;R^{*}\left(t\right),\;V^{*}\left(t\right),\;K^{*}\left(t\right)\right)$ corresponds to a positive ω -periodic solution of model (1). Thus, the proof is complete.

## 4 A case study: Influenza transmission in Chinese provinces

In this section, we fit the unknown parameters in model (1) using data from various provinces and regions in China during 2023–2024. This includes influenza cases, BSI values, and meteorological data, specifically average temperature, total precipitation, and relative humidity. Based on these fitted parameters, we infer the effective reproduction number for each province and region.

### 4.1 Data collection and description

We collected the number of influenza cases during 2023–2024 from the Centers for Disease Control and Prevention or Health Commissions of Anhui Province, Gansu Province, Henan Province, Chongqing Municipality, Shanghai Municipality, Sichuan Province, Zhejiang Province, and the Xinjiang Uygur Autonomous Region in China. For example, data for Anhui Province were obtained from the Anhui Provincial Center for Disease Control and Prevention [24]. Fig A1 in Appendix presents the influenza case numbers for different provinces.

The 2023–2024 BSI data and corresponding regions are from the Baidu Index platform [25]. To construct a representative measure of public interest and information-seeking behavior related to seasonal influenza, we selected four keywords that are closely associated with flu-related symptoms and terminology: “cough”, “fever”, “cold” and “influenza” [26]. The composite BSI was generated by aggregating the search volumes of these four keywords, providing a proxy for media coverage and public awareness in each province. Fig A1 in Appendix presents the BSI for different provinces.

**Fig 2:**
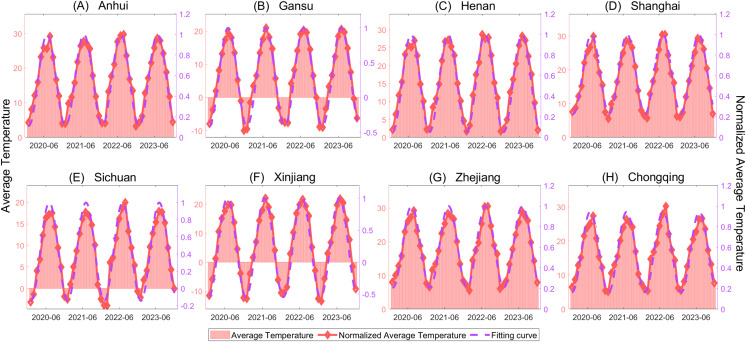
Fitting results of monthly average temperature for representative provinces from 2020 to 2023. The bar charts represent the observed average temperature values, the red lines indicate the normalized temperature series, and the purple dashed lines denote the fitted curves.

**Fig 3:**
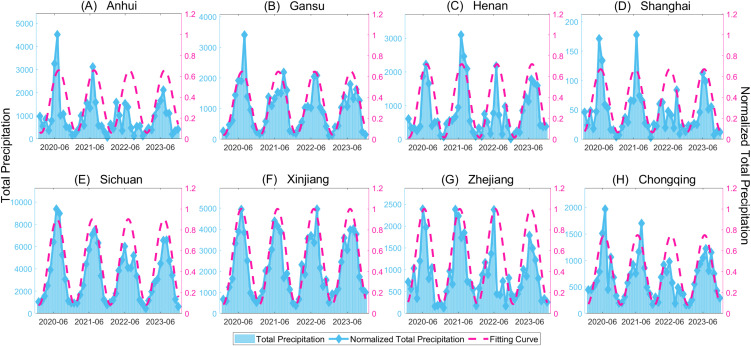
Fitting results of total monthly precipitation for representative provinces from 2020 to 2023. The bar charts show the observed total precipitation (calculated by summing the monthly precipitation across all grid points within each provincial region); the blue lines represent the normalized precipitation series, and the pink dashed lines indicate the fitted curves.

The meteorological data used in this study are derived from the ERA5 reanalysis dataset (https://cds.climate.copernicus.eu) and include monthly average temperature (MAT), monthly average relative humidity (MARH), and monthly total precipitation (MTP) [27]. These data have a spatial resolution of 0.5° × 0.5° and cover the period January 2020 to December 2023. For each province, monthly average temperature and relative humidity were computed by averaging values over all grid points within the provincial boundary, while monthly total precipitation was obtained by summing precipitation across those same grid cells. The resulting provincial series are presented in Figs 2 and 3.

### 4.2 Preliminary data analysis

To investigate the relationship between influenza activity and potential influencing factors, we analyzed the correlation between the BSI and influenza case numbers. The results indicated a statistically significant positive correlation between the two variables during the period from 2023 to 2024 (see Appendix, Fig A2), suggesting that the BSI effectively reflects public awareness of influenza. Further analysis at the provincial level revealed that the BSI and influenza cases generally exhibited a strong and significant positive correlation across the provinces and regions. For instance, in Anhui Province, the correlation coefficient reached 0.90 (p<0.01). These findings overall support the validity of using the BSI as a proxy indicator for public attention to influenza.

**Fig 4:**
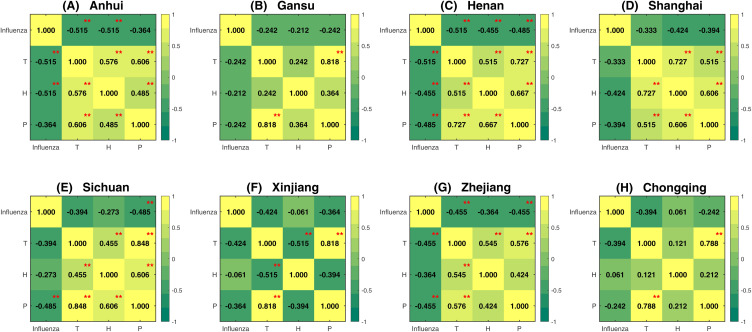
Kendall’s rank correlation coefficients between influenza incidence and various meteorological factors across different provinces in 2023. T, H, and P represent MAT, MARH and MTP, respectively. Red star marks statistically significant correlations (p<0.05).

Kendall’s rank correlation coefficient is employed to assess the relationship between influenza cases and meteorological factors across Chinese provinces. As shown in Fig 4, influenza cases generally exhibit negative correlations with meteorological factors. Using Zhejiang Province (a coastal region) and the Xinjiang Uygur Autonomous Region (an inland northwestern area) as representative examples, monthly average temperature and total monthly precipitation show the strongest associations with influenza cases, whereas the correlation with monthly average relative humidity is relatively weaker. Therefore, only temperature and precipitation are included as key meteorological covariates in the subsequent modeling.

To capture the seasonal patterns of these variables, sinusoidal functions are used for fitting. All meteorological data are standardized to account for differences in measurement units and improve comparability. The fitting is conducted using the least squares method, with results presented in Figs 2 and 3.

Figs 2 and 3 present the fitting performance for monthly average temperature and total monthly precipitation, respectively. The general forms of the corresponding fitting functions are given as follows,


$$\begin{array}{c} \begin{array}{c} T\left(t\right)=T_{0}+T_{1}sin(\frac{\pi }{6}t+T_{\varphi }),\\ P\left(t\right)=P_{0}+P_{1}sin(\frac{\pi }{6}t+P_{\varphi }), \end{array} \end{array}$$ (22)

where π/6 in equations (22) represents the periodicity of 12 months. The estimated parameter values for each province are summarized in Table B1 of Appendix.

### 4.3 Estimation of model parameters

In this section, we focus on estimating the parameters of the proposed model. At present, the exact functional forms of the viral clearance rate ρ(t) and the transmission rate β(t) remain undetermined. Initially, regarding the viral clearance rate, as mentioned in the Introduction, the clearance of influenza viruses is affected by temperature and precipitation. Handel et al. [9] described the temperature-dependent decay rate of influenza viruses in the environment as $\delta \left(T\right)=\alpha e^{\gamma T},$ where T represents temperature, and α and γ are constants. Subsequently, Jing et al. [7] extended this formulation to account for the effects of additional meteorological factors on viral decay. Based on these considerations, the viral clearance rate in our model is defined as


$$\begin{array}{c} \rho \left(t\right)=\rho_{0}\;e^{\alpha_{1}\;T\left(t\right)+\alpha_{2}\;P\left(t\right)}, \end{array}$$

where $\rho_{\text{0}}\;$ is the baseline clearance rate, T denotes temperature, P represents precipitation, and $\alpha_{1}$ and $\alpha_{2}$ are parameters to be estimated.

To estimate the temporal variation of the transmission rate β(t), we employ a random sampling approach based on the Markov Chain Monte Carlo (MCMC) algorithm for parameter estimation in model (1) [28]. Specifically, β(t) is represented as a time-dependent function with a smooth structure, and its implicit form is constructed using spline functions. To improve sampling efficiency and acceptance rates in complex models, we use the Delayed Rejection Adaptive Metropolis (DRAM) algorithme [29], which optimizes the MCMC parameter estimation process. On this basis, the estimates of β(t) at each time point are obtained through MCMC sampling. Additionally, the temporal trajectory of β(t) is fitted using a truncated Fourier series, expressed as


$$\begin{array}{c} \beta \left(t\right)=a_{0}\;+\overset{n}{\underset{1}{\sum }}\left(a_{n}\;cos\left(nwt)+b_{n}\;sin(nwt\right)\right),\;n=2. \end{array}$$

thereby extracting the explicit periodic structure of the transmission rate.

All parameters and initial values in model (1) were estimated. Some were directly determined based on publicly available data, as detailed below,

(i) Recruitment rate (Λ): This rate is obtained from the Bureau of Statistics or official government websites of each province. Specifically, the annual number of births is calculated by multiplying the resident population at the end of 2022 by the birth rate, and then dividing by 12 to yield the monthly recruitment rate. For example, in Henan Province, the annual number of births is 695,000, resulting in Λ≈57,917 per month [30].
(ii) Natural mortality rate (d): This rate is derived from the average life expectancy reported by provincial statistical agencies and is calculated as $\begin{array}{c} d=1/\left(\text{life\textbackslash{} expectancy}\times 12\right) \end{array}$. For instance, the life expectancy in Henan Province in 2023 is 78.03 years, giving d=1/(78.03×12) [31].
(iii) Recovery rate of infected individuals (η): According to the World Health Organization [1] and related literature [32], most influenza patients recover naturally within 7 days. Therefore, the recovery rate is assumed to be η=30/7.
(iv) Initial value of influenza cases (I(0)): This is determined based on the number of influenza cases in January 2023 from provincial Health Commissions. For Henan Province, I(0)=1322 [33].
(v) Initial value of the BSI (K(0)): This is taken from the BSI for influenza in January 2023. For Henan Province, K(0)=5212 [25].
(vi) The province-specific estimation of the remaining parameters and initial conditions in the model is performed using the MCMC method. The corresponding estimation results are detailed in Tables B2 and B3 of Appendix.

**Fig 5:**
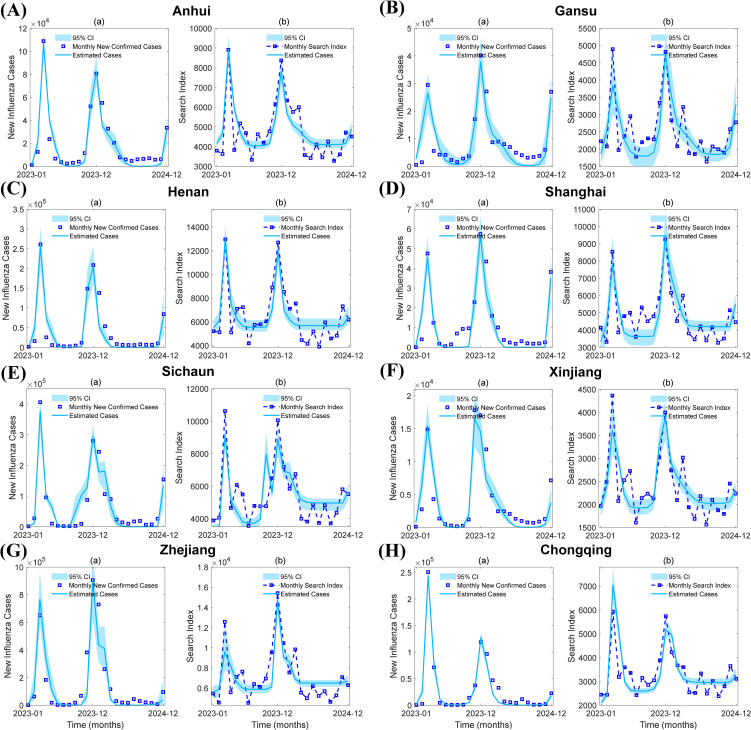
Model fitting using influenza case data and BSI data. (**a)** Model fitting based on influenza case data from several provinces. The blue solid line represents the model estimates, and the light blue shaded area denotes the 95% confidence interval. **(b)** Model fitting using BSI data. The blue solid line represents the model estimates, and the light blue shaded area denotes the 95% confidence interval.

**Fig 6:**
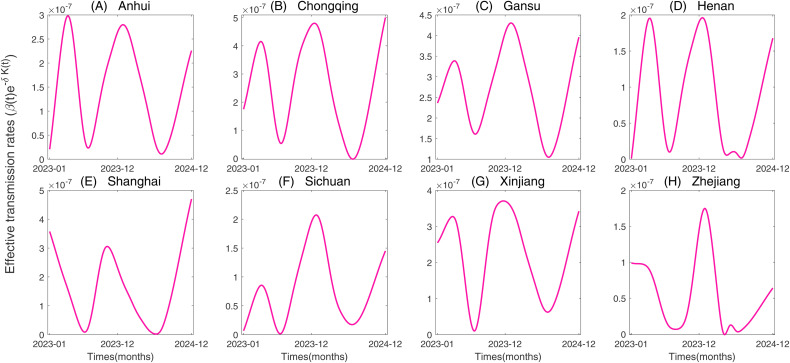
Effective transmission rates $\beta \left(t\right)e^{-\delta K\left(t\right)}$ over time (2023–2024) for different provinces in China. $\begin{array}{c} \beta \left(t\right)e^{-\delta K\left(t\right)} \end{array}$ represents the effective transmission rate suppressed by the media-driven public awareness of the transmission rate.

**Fig 7:**
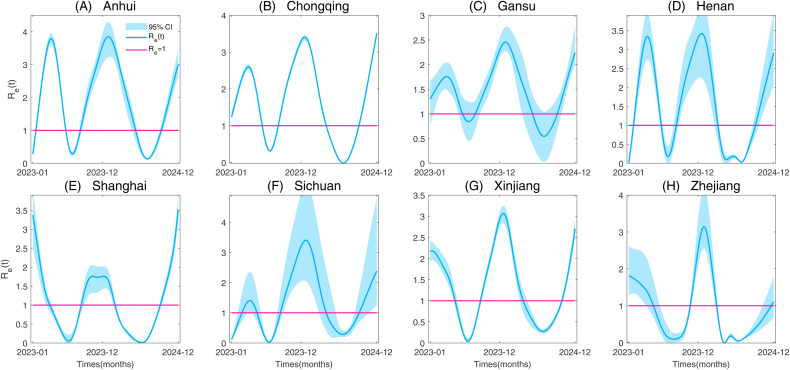
Effective reproduction numbers $R_{e}\left(t\right)$ for each province from 2023 to 2024. The blue curve represents the effective reproduction number, while the blue shaded area indicates the 95% confidence interval. The pink horizontal line represents the threshold where $\begin{array}{c} R_{e}\left(t\right) \end{array}$ equals 1.

Figs 5–7 present the model fitting and estimation of key epidemiological parameters for different provinces in China. Fig 5 shows the model fit to the actual influenza case data and the BSI data. Fig 6 illustrates the effective transmission rates over time, highlighting the impact of media-driven public awareness on reducing transmission rates. Fig 7 depicts the temporal changes in the effective reproduction number $R_{e}\left(t\right)$ for each province.

## 5 Sensitivity analysis

Seasonal influenza outbreaks are influenced by a variety of factors, including ambient temperature, total precipitation, and media-driven public awareness and others. In this section, we perform a sensitivity analysis to evaluate how these factors affect the number of new influenza cases and the effective reproduction number $R_{e}\left(t\right)$ across different provinces.

### 5.1 Sensitivity analysis of media-driven public awareness

In this section, we explore the influence of media-driven public awareness on influenza transmission dynamics, specifically its effects on new influenza cases and the effective reproduction number $R_{e}\left(t\right)$. To examine this impact, we vary the parameter δ by ±20% and analyze the consequent effects on the 2024 influenza cases and $R_{e}\left(t\right)$.

**Fig 8:**
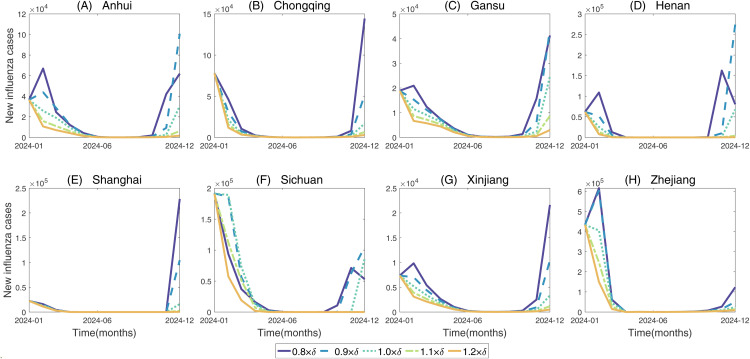
Impact of media-driven public awareness fluctuations on influenza transmission trends in different provinces of China in 2024.

**Fig 9:**
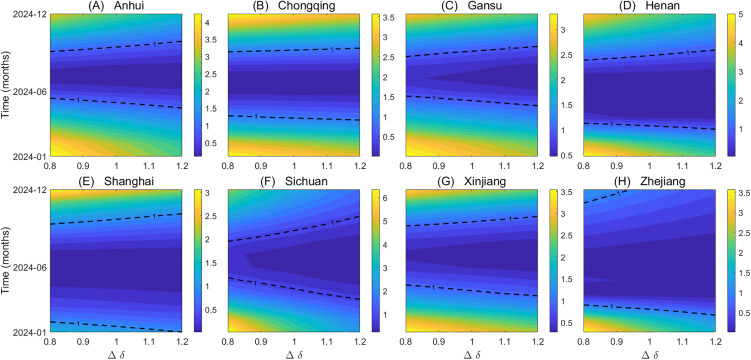
Impact of media-driven public awareness fluctuations on the effective reproduction number of influenza across different provinces in China in 2024. The black dashed line indicates the contour corresponding to $R_{e}\left(t\right)=1$, and the color bar represents the value of $R_{e}\left(t\right)$.

Fig 8 demonstrates that under strong media influence (i.e., higher δ values), new influenza cases are significantly reduced, particularly during peak periods, underscoring the potential of media-driven public awareness as an effective non-pharmaceutical intervention. Fig 9 illustrate the impact of media-driven public awareness fluctuations on the $R_{e}\left(t\right)$ of influenza transmission across various provinces in China in 2024. The results indicate that reducing the media influence parameter δ, for example by 20%, results in a noticeable increase in $R_{e}\left(t\right)$, especially during peak transmission periods. For example, in Anhui Province, a 20% decrease in δ results in an increase of approximately 0.75 in $R_{e}\left(t\right)$ by the end of 2024, corresponding to a 27.5% rise. Overall, Sichaun, Zhejiang, Henan and Anhui demonstrate greater sensitivity to media-driven awareness fluctuations, as evidenced by increases in $R_{e}\left(t\right)$ of approximately 61.3%, 46.8%, 30.3%, and 27.5%, respectively, following a 20% reduction in δ. By contrast, Xinjiang, Gansu, and Chongqing show relatively weaker responses, with increases all below 20%. The weaker responsiveness observed in some inland regions is likely driven by a combination of structural factors, rather than any single determinant. These may include differences in information infrastructure, socioeconomic development, public risk perception, and regional behavioral patterns, which jointly influence the effectiveness of media-driven awareness (see Appendix E in the Supplementary Materials).

### 5.2 Sensitivity analysis of MAT and MTP

**Fig 10:**
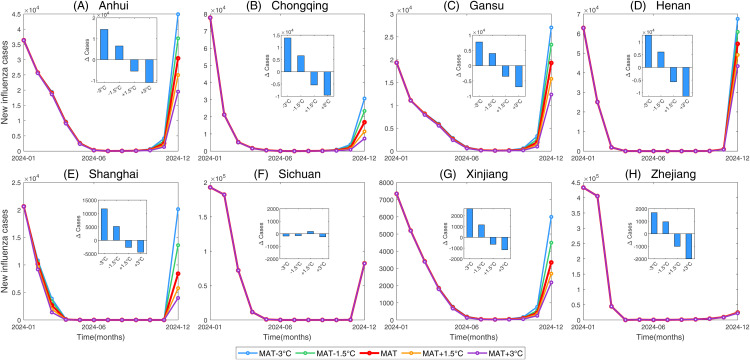
Impact of MAT perturbations (–3 °C, –1.5 °C, baseline, + 1.5 °C, + 3 °C) on new influenza cases across provinces in China in 2024. Main panels show simulated time series under each MAT scenario; insets present changes in new cases at the end of 2024 relative to the baseline.

This section examines the sensitivity of influenza cases and the effective reproduction number to variations in temperature and total precipitation across provinces. As shown in Fig 10, perturbations in monthly average temperature (+3 °C, + 1.5 °C, –1.5 °C, –3 °C) substantially affect the intensity of epidemic peaks. For example, in Anhui, a 3 °C decrease results in over 14,000 additional new cases by late 2024 (a 47.48% increase), whereas a 3 °C increase leads to more than 10,000 fewer cases (a 36.01% reduction) relative to the baseline. These findings highlight the strong modulatory role of temperature in influenza transmission, primarily through its effects on viral environmental stability and human contact behavior.

**Fig 11:**
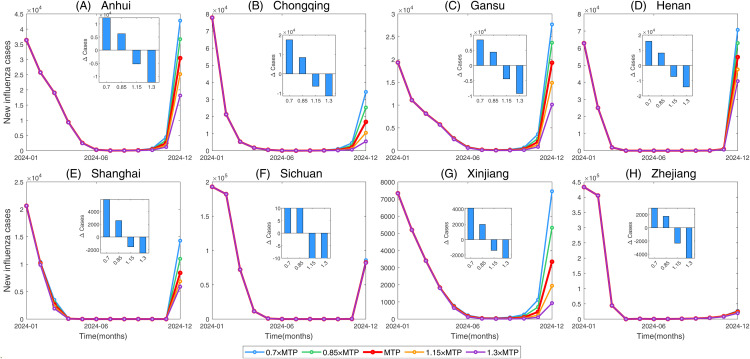
Impact of MTP perturbations (±30%) on new influenza cases across different provinces in China in 2024. The main panels show the time series of new cases under each MTP scenario, while the inset plots present the changes in new cases by the end of 2024 relative to the baseline.

By comparing Figs 10 and 11, it can be further observed that precipitation perturbations also have a significant impact on the intensity of influenza peaks, effectively regulating the strength of virus transmission. It can be seen that the impact of precipitation perturbations shows considerable heterogeneity across regions, and the underlying mechanisms depend more on local climate conditions and human behavioral patterns. For example, in Xinjiang, a predominantly arid and semi-arid region, variations in monthly total precipitation have a more marked impact on influenza incidence. This may be attributed to the fact that in such dry environments, precipitation-induced changes in ambient humidity can significantly affect aerosol transmission efficiency, thereby amplifying the influence of precipitation perturbations on the transmission dynamics.

**Fig 12:**
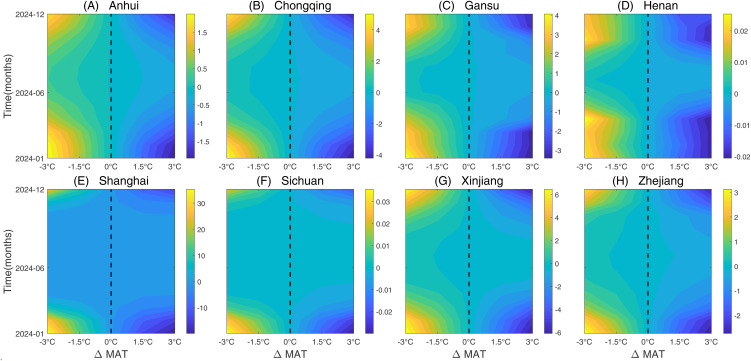
Relative changes in the effective reproduction number of influenza in 2024 across various provinces in China, under MAT perturbations (−3 °C, −1.5 °C, + 1.5 °C, and +3 °C), compared to the baseline. The black dashed line represents the baseline scenario, and the color bar represents the relative change of $\begin{array}{c} R_{e}\left(t\right) \end{array}$ compared to the baseline.

In the temperature perturbation simulations shown in Fig 12, winter temperature changes significantly affect the effective reproduction number. When the MAT decreases by 3°C, the peak of $R_{e}\left(t\right)$ in most provinces is notably higher than in the baseline scenario, indicating that colder conditions enhance influenza transmission potential. For instance, in Shanghai, when MAT is reduced by 3°C, the peak $R_{e}\left(t\right)$ up to December 2024 is about 27.33% higher than the baseline. This change is likely due to the longer survival time of the virus in colder environments and increased indoor activities, which raise contact rates in enclosed spaces. In contrast, under the MAT + 3°C scenario, Shanghai’s peak $R_{e}\left(t\right)$ up to December 2024 decreases by approximately 13.16%, suggesting that higher temperatures disrupt the transmission chain, thereby reducing epidemic intensity.

**Fig 13:**
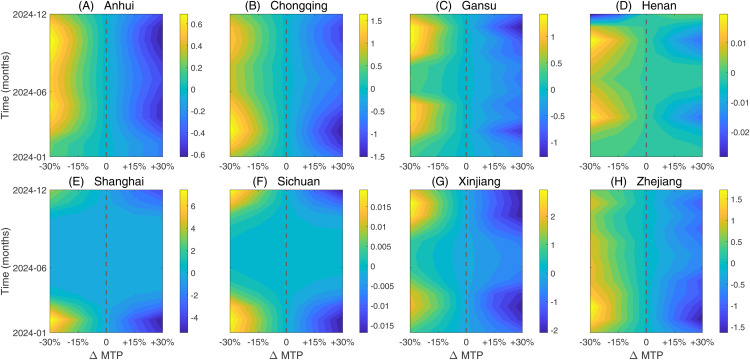
Relative changes in the effective reproduction number of influenza in 2024 across various provinces in China under MTP perturbations (±30%), compared to the baseline. The black dashed line represents the baseline scenario, and the color bar represents the relative change of $\begin{array}{c} R_{e}\left(t\right) \end{array}$ compared to the baseline.

Fig 13 illustrates the variation in influenza $R_{e}\left(t\right)$ under different MTP perturbation scenarios. Overall, the impact of precipitation is more pronounced in arid or semi-arid regions of the northwest and in certain coastal metropolitan areas. For example, in Xinjiang and Gansu, a 30% reduction in MTP leads to a significant rise in $R_{e}\left(t\right)$ during the epidemic season. This effect may stem from the enhanced efficiency of aerosol transmission and reduced viral sedimentation rates in dry environments, which extend the airborne survival and travel distance of the virus. In coastal and urban areas, short-term fluctuations in relative humidity induced by precipitation may interact with behavioral factors such as indoor crowding and ventilation changes, further amplifying the transmission response to precipitation perturbations [34].

### 5.3 Global sensitivity analysis of the effective reproduction number

In this section, we conducted a global sensitivity analysis of the effective reproduction number $R_{e}\left(t\right)$ using the Latin Hypercube Sampling–Partial Rank Correlation Coefficient (LHS-PRCC) method [35]. We generated 20,000 parameter sets using Latin Hypercube Sampling within ±15% of the baseline values listed in Table B2 and B3 of Appendix, ensuring adequate coverage of the high-dimensional parameter space. For each parameter set, the corresponding $R_{e}\left(t\right)$ value was computed, and PRCC was applied to quantify the independent correlation between each input parameter and $R_{e}\left(t\right)$, controlling for the influence of other variables.

**Fig 14:**
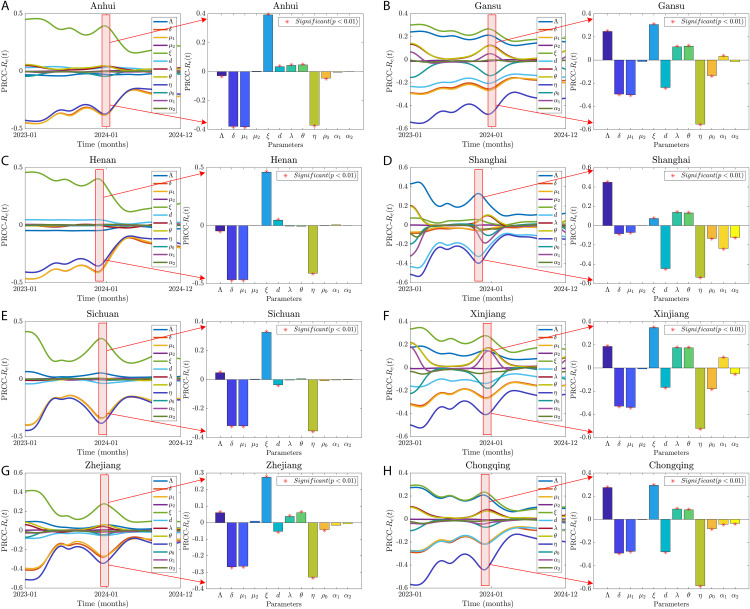
PRCC values of the $R_{e}\left(t\right)$ across provinces. The left panels show the temporal trends of PRCC values between parameters and $\begin{array}{c} R_{e}\left(t\right) \end{array}$, while the right panels present the PRCC values and their statistical significance around early 2024 when parameter influence peaks.

Columns 1 and 3 of Fig 14 illustrate the temporal variation in the influence of different parameters on $R_{e}\left(t\right)$ across provinces, as measured by PRCC values. Overall, the patterns of parameter influence on $R_{e}\left(t\right)$ are broadly consistent across provinces. However, these effects are not constant over time, exhibiting clear temporal dynamics. During the peak of influenza outbreaks (winter), the influence of most parameters on $R_{e}\left(t\right)$ becomes more pronounced, whereas during the summer months, as transmission intensity declines, their impact gradually diminishes.

Columns 2 and 4 of Fig 14 present the PRCC values and their statistical significance (p<0.01) for different parameters with respect to $R_{e}\left(t\right)$ across provinces around early 2024, when the parameter influence peaks. Overall, in most provinces during this period, parameters ξ, Λ, λ and θ show significant positive associations with $R_{e}\left(t\right)$, while parameters η,δ,$\mu_{1},$d,$\rho_{0},$ and $\alpha_{1}$ exhibit negative associations. These results highlight systematic differences in how various types of parameters regulate the transmission potential of influenza during peak epidemic periods.

It can be observed that increases in media and behavior-related parameters $\mu_{1}$ and δ, along with a decrease in ξ, are associated with a significant reduction in $R_{e}\left(t\right),$ indicating that these parameters play a suppressive role in influenza transmission. Notably, in Shanghai, the positive association between the natural dissipation rate of media influence ξ and the effective reproduction number $R_{e}\left(t\right)$ is considerably weaker than in other provinces. This finding indicates a lower sensitivity of influenza transmission to media information decay in this region, implying that protective behaviors remain stable despite the gradual weakening of media influence, which in turn helps to suppress transmission intensity.

The meteorological parameters $\rho_{0}$, $\alpha_{1}$ and $\alpha_{2}$ are generally negatively correlated with $R_{e}\left(t\right)$. This indicates that higher values of these parameters correspond to increased viral clearance from the air, resulting in lower airborne viral concentrations and, consequently, reduced transmission potential. Moreover, the commonly observed pattern of $\begin{array}{c} \left|\text{PRCC}\left(\alpha_{1}\right)\right|>\left|\text{PRCC}\left(\alpha_{2}\right)\right| \end{array}$ further supports the notion that temperature has a more pronounced impact on viral clearance compared to total precipitation.

However, an exception is observed in the northwestern provinces of Gansu and Xinjiang, where $\alpha_{1}$ has a positive effect on $R_{e}\left(t\right)$. Here, $\alpha_{1}$ represents the influence of temperature variation, and an increase in $\alpha_{1}$ leads to a rise in the virus clearance rate ρ(t). This seemingly paradoxical result is largely attributable to the region’s extreme climatic conditions. During winter, temperatures in Xinjiang and Gansu often remain around –10°C (see Fig 3). Under such conditions, even a moderate increase in temperature may still fall within a low absolute range, insufficient to significantly enhance viral inactivation. As a result, the expected effect of improved viral clearance associated with higher $\alpha_{\text{1}}$ is not realized.

Moreover, the LHS-PRCC method evaluates global sensitivity by accounting for simultaneous variations of parameters within a multidimensional space, which differs from the direct causal effects revealed through univariate perturbations (as discussed in Section 5.2). The positive PRCC values for $\alpha_{\text{1}}$ observed in Gansu and Xinjiang may reflect region-specific interactions between temperature sensitivity and other epidemiological parameters or behavioral factors. This further underscores the complex regulatory role of climatic factors in shaping influenza transmission dynamics, particularly in regions with extreme weather conditions, where such mechanisms may exhibit nonlinear and region-specific responses.

In Fig C1 of Appendix, we illustrate the temporal dynamics of the statistical significance of each parameter’s association with $R_{e}\left(t\right)$. The results indicate that parameters η, δ,ξ and $\mu_{1}$ are significantly correlated with $R_{e}\left(t\right)$ across most time points, underscoring their consistently important roles in regulating viral transmission. By contrast, parameters such as λ,θ,$\rho_{0},$$\alpha_{1}$ and $\alpha_{2}$ show statistical significance primarily during the winter influenza peak.

## 6 Forecasting future trends of influenza

**Fig 15:**
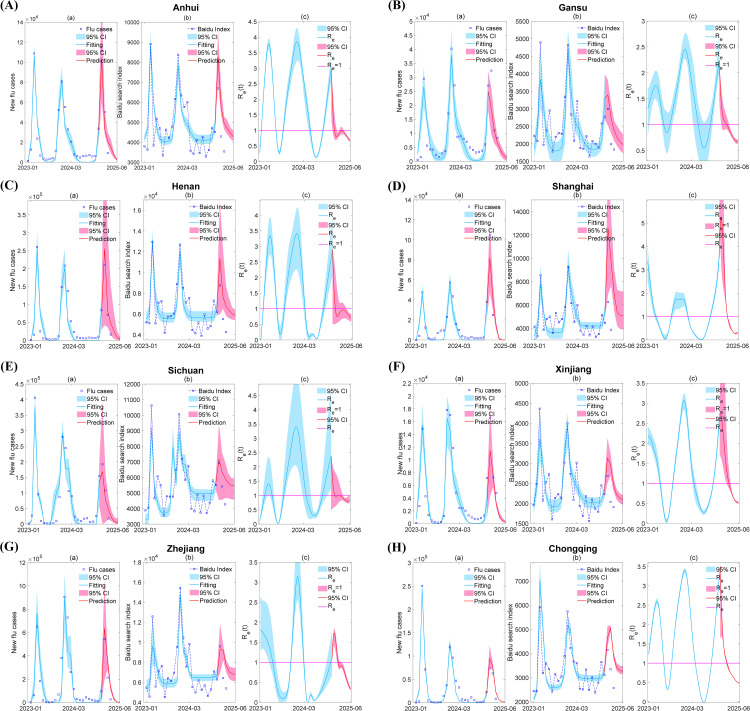
Forecasts of new influenza cases, BSI, and effective reproduction number for each province over the next six months. The red line shows the predicted mean, and the red shaded area shows the 95% confidence interval. Blue squares represent the observed data.

To assess the future trajectory of influenza transmission in China, we simulated the dynamics of influenza cases in 2025 based on the parameter estimates and confidence intervals obtained in Section 4. By integrating province-specific parameters and environmental inputs, we forecast the number of new influenza cases, BSI trends, and the evolution of the effective reproduction number over the next six months. To evaluate predictive performance, influenza case data and BSI records from 2025 serve as a validation set. The corresponding visualizations are shown in Fig 15.

Fig 15 provides detailed forecasts of influenza activity over the next six months. Overall, the model demonstrates reasonable accuracy in reproducing seasonal transmission patterns and regional differences. Most provinces are projected to experience an increase in influenza activity in early 2025, with varying peak intensities. Notably, Anhui, Henan, and Shanghai are expected to exhibit the most pronounced surges in case numbers, accompanied by periodic fluctuations in $R_{e}\left(t\right)$. In contrast, provinces such as Chongqing and Sichuan show relatively lower and more stable levels of transmission.

The predicted values for influenza cases closely match the reported data across provinces. Forecasts of the BSI also align well with observed trends in several provinces, although overestimations are noted in a few regions. Particularly in Sichuan, Gansu, and Zhejiang, where the predicted mean trends are relatively close to the observed values. Deviations were observed in Anhui, Shanghai and other provinces, and the predicted peak value of the search index was higher than the validation data. Nevertheless, the overall trend has been well grasped.

This combination of qualitative and quantitative evaluation suggests that the model effectively captures broad transmission patterns and key epidemic dynamics. However, regional heterogeneity and variability in public response introduce some uncertainty into short-term forecasts [36].

## 7 Conclusion and discussion

In this study, we developed a non-autonomous influenza transmission model by extending the traditional *SIR* framework to incorporate environmental viral concentration and media-driven public awareness, with meteorological factors embedded into the viral clearance rate. Theoretical analysis established the model’s positive invariant set and time-varying effective reproduction number. Furthermore, it was shown that the disease-free periodic solution is globally asymptotically stable and that the model exhibits uniform persistence. Model parameters were estimated using provincial influenza case data, Baidu search indices, and ERA5 reanalysis data through MCMC methods. This estimation enabled the reconstruction of time-varying effective transmission rates and effective reproduction numbers across different regions. Sensitivity analyses were performed to systematically assess the impact of key factors, such as media campaigns and meteorological conditions, on influenza transmission dynamics.

Our findings highlight that enhanced media influence significantly suppresses influenza incidence during peak seasons, underscoring the value of media campaigns as a non-pharmaceutical intervention. Furthermore, the degree of sensitivity to media influence exhibited regional variation: provinces including Anhui, Sichuan, Zhejiang, and Henan showed pronounced responsiveness, whereas Xinjiang, Gansu, and Chongqing exhibited limited sensitivity. These observations imply that timely, continuous media interventions are especially critical for outbreak mitigation in regions highly susceptible to changes in public awareness.

Perturbation analyses of meteorological variables revealed that decreases in MAT enhanced viral transmissibility, resulting in more sharply peaked winter epidemics, while increases in MAT suppressed transmission intensity. For instance, a 3 °C decrease in MAT led to a 47.48% increase in new cases in Shanghai relative to the baseline by late 2024, whereas a 3 °C increase reduced cases by 36.01%. MTP perturbations also exhibited substantial regional heterogeneity: in arid regions such as Xinjiang and Gansu, a 30% reduction in MTP considerably exacerbated epidemic severity, likely reflecting enhanced aerosol transmission efficiency under low-humidity conditions. Global sensitivity analysis further revealed that temperature has a stronger and generally negative impact on the effective reproduction number compared to precipitation, with a positive correlation in most regions and a negative correlation in arid and semi-arid areas.

By integrating key driving factors into a unified predictive framework, we projected influenza activity in early 2025 and validated the model using newly reported cases and search index data, demonstrating a high degree of consistency. It should be noted that these projections are conditional on the continuation of historical meteorological patterns rather than explicit forecasts of future climate conditions. These findings underscore that combining meteorological forecasting with regionally targeted media interventions can improve the accuracy of influenza activity predictions and inform more effective non-pharmaceutical response strategies. More broadly, this study elucidates the complex, nonlinear interactions among meteorological conditions, human behavior, and information dissemination in shaping epidemic dynamics, offering actionable insights for public health policy and preparedness. Nevertheless, uncertainties in future climate variability may influence the magnitude and timing of transmission, and incorporating scenario-based or ensemble meteorological inputs represents an important direction for future research.

## Supporting information

S1 Appendix Supporting Information-Appendix. (PDF)
