# Supplementary material for: Modeling the effects of meteorological factors and media-driven public awareness on seasonal influenza outbreaks
Source: PLoS One. 2026 Jun 8;21(6):e0342962. doi: 10.1371/journal.pone.0342962 (PMC13245874; doi:10.1371/journal.pone.0342962)
Supplement: S1 Appendix — (PDF) [file pone.0342962.s001.pdf]

Appendix A

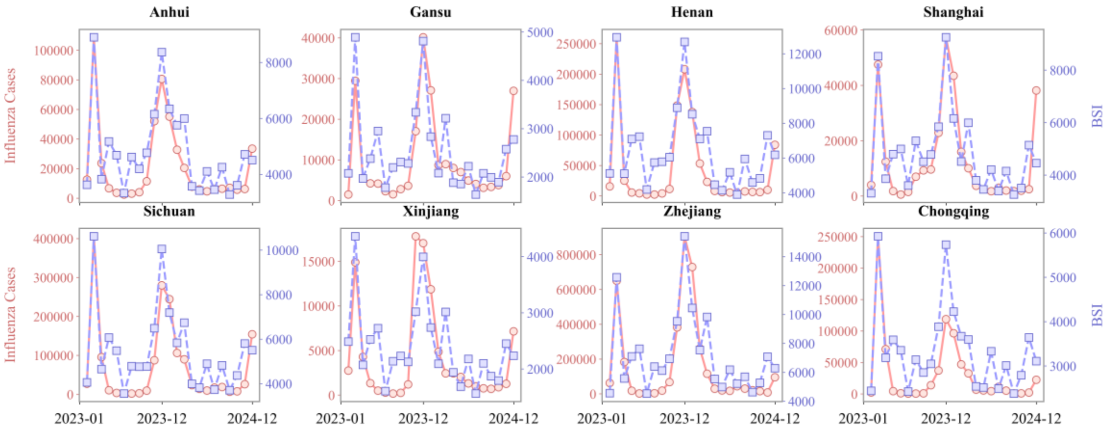

Fig. A1 Trend of influenza cases and BSI across different provinces of China (2023-2024). The blue square line represents influenza cases, and the red circular line represents the BSI.

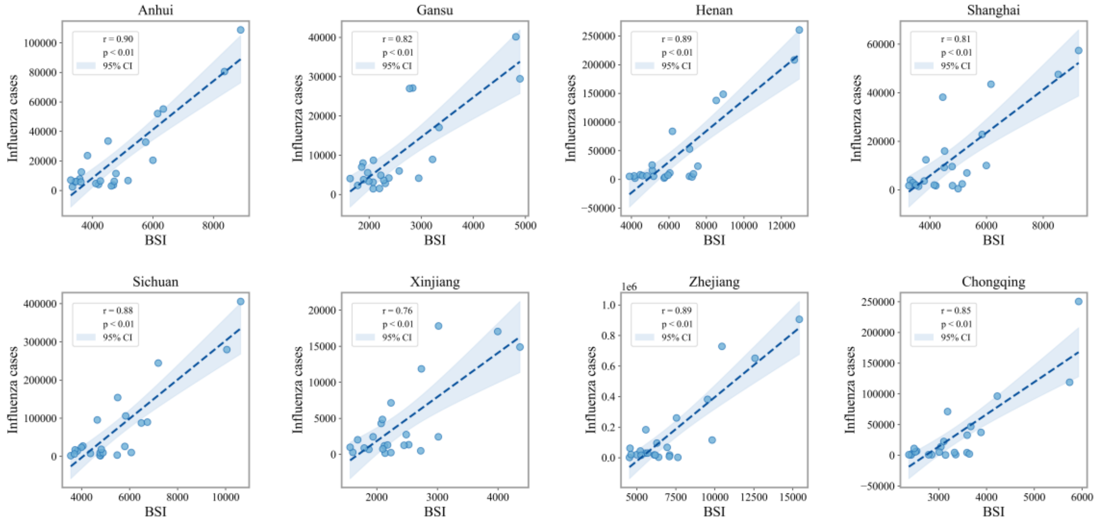

Fig. A2 Correlation analysis between influenza cases and BSI across different provinces of China (2023-2024).

Appendix B

Table B1 Estimated parameters for temperature and precipitation across provinces

Parameters	Anhui	Gansu	Henan	Shanghai	Sichuan	Xinjiang	Zhejiang	Chongqing
T_0	0.5554	0.2937	0.5258	0.6148	0.4281	0.2264	0.6048	0.5547
T_1	0.4446	0.7063	0.4742	0.3852	0.5719	0.7736	0.3952	0.3899
T_φ	-2.1279	10.5000	-2.0673	-2.2618	-2.1465	10.6000	-8.4906	-2.1405
P_0	0.3580	0.3448	0.3683	0.3685	0.4918	0.5428	0.5446	0.4152
P_1	0.2998	0.3037	0.3524	0.3030	0.4098	0.4572	0.4554	0.3334
P_φ	-8.6048	4.0733	-2.2115	-1.8492	-2.1767	10.5000	-8.0906	-1.8849

Table B2(1) Parameter estimates and initial values of the model (1)

Parameters	Anhui	Gansu	Henan	Shanghai
d	1/(80.72×12)	1/(76.64×12)	1/(78.03×12)	1/(83.18×12)
Λ	36500	15917	57917	8167
θ	0.000908	0.003746	9.92E-05	0.012356
ξ	0.812249	0.638444	0.792567	1.822496
μ_1	7.32E-05	7.12E-05	6.07E-05	1.39E-04
μ_2	0.159745	0.331595	0.082551	0.471558
μ_3	0.000411	0.000913	0.000317	0.005854
α_1	1.24877	1.298016	2.126779	7.070435
α_2	1.350799	1.245256	2.060505	5.72141
ρ_0	0.084489	0.143669	0.01104	0.028033
δ	0.000337	0.000321	0.000269	0.000215
λ	12.36378	8.788704	0.341127	11.86578
S_0	54208991	23684478	89453645	23527385
R_0	6856	5182	16094	6127
V_0	8969	4549	75904	1811

Table B2(2) Parameter estimates and initial values of the model (1)

Parameters	Sichuan	Xinjiang	Zhejiang	Chongqing
d	1/(78.5×12)	1/(76×12)	1/(82.4×12)	1/(79.39×12)
Λ	44083	14080	31917	14900
θ	2.04E-05	0.004307	0.000365	0.00263
ξ	1.224388	0.858374	1.415833	0.526047
μ_1	4.29E-05	9.11E-05	9.41E-05	8.16E-05
μ_2	0.051011	0.488763	0.025249	0.096138
μ_3	0.000876	0.002342	0.000366	0.001539
α_1	1.613594	1.640143	1.805183	1.607272
α_2	5.653342	1.21214	1.567984	1.731687
ρ_0	0.025499	0.066554	0.003825	0.039543
δ	0.000627	0.000448	0.00038	0.000218
λ	2.062985	5.901874	3.252286	5.174034
S_0	75047118	20179236	59273804	25187027
R_0	9318	12448	4253	5839
V_0	5073	6164	50523	13472

Table B3(1) Parameter Estimates of $\beta(t)$ with 95% CI

Parameters	Anhui		Gansu	
	Value	95% CI	Value	95% CI
a_0	4.67E-07	[3.83E-07, 5.51E-07]	5.15E-07	[4.84E-07 5.47E-07]
a_1	-1.41E-07	[-2.82E-07 1.72E-10]	-9.08E-08	[-1.47E-07 -3.47E-08]
b_1	7.04E-08	[-8.25E-08 2.23E-07]	2.53E-08	[-3.38E-08 8.44E-08]
a_2	1.13E-07	[-1.67E-07 3.92E-07]	1.27E-07	[1.60E-08 2.38E-07]
b_2	2.83E-07	[1.24E-07 4.41E-07]	1.77E-07	[9.55E-08 2.59E-07]
w	0.2732	[0.2409 0.3055]	0.2732	[0.2524 0.294]

Table B3(2) Parameter Estimates of $\beta(t)$ with 95% CI

Parameters	Henan		Shanghai	
	Value	95% CI	Value	95% CI
a_0	3.61E-07	[2.67E-07 4.54E-07]	3.14E-07	[2.65E-07 3.63E-07]
a_1	-9.58E-08	[-3.15E-07 1.24E-07]	6.85E-08	[-3.30E-08 1.70E-07]
b_1	1.30E-07	[-2.55E-08 2.85E-07]	9.05E-08	[2.88E-08 1.52E-07]
a_2	1.82E-07	[-1.01E-07 4.65E-07]	2.96E-07	[2.31E-07 3.62E-07]
b_2	1.72E-07	[-1.17E-07 4.61E-07]	5.57E-08	[-1.23E-07 2.35E-07]
w	0.2732	[0.2221 0.3243]	0.2732	[0.2509 0.2955]

Table B3(3) Parameter Estimates of $\beta(t)$ with 95% CI

Parameters	Sichuan		Xinjiang	
	Value	95% CI	Value	95% CI
a_0	1.04E-06	[8.51E-07 1.23E-06]	5.74E-07	[5.15E-07 6.32E-07]
a_1	-5.44E-07	[-8.69E-07 -2.18E-07]	-9.59E-08	[-2.10E-07 1.83E-08]
b_1	-1.45E-07	[-6.31E-07 3.42E-07]	4.58E-08	[-5.07E-08 1.42E-07]
a_2	6.89E-07	[4.19E-08 1.34E-06]	2.72E-07	[8.52E-08 4.58E-07]
b_2	4.24E-07	[-3.97E-07 1.25E-06]	2.43E-07	[4.87E-08 4.36E-07]
w	0.2732	[0.2254 0.321]	0.2732	[0.2477 0.2987]

Table B3(4) Parameter Estimates of $\beta(t)$ with 95% CI

Parameters	Zhejiang		Chongqing	
	Value	95% CI	Value	95% CI
a_0	3.89E-07	[3.00E-07 4.78E-07]	4.23E-07	[3.61E-07 4.86E-07]
a_1	-1.04E-07	[-2.61E-07 5.42E-08]	-9.44E-08	[-2.32E-07 4.33E-08]
b_1	5.10E-08	[-9.77E-08 2.00E-07]	9.95E-08	[-2.99E-09 2.02E-07]
a_2	2.37E-07	[-6.47E-08 5.39E-07]	2.27E-07	[3.52E-08 4.20E-07]
b_2	3.36E-07	[1.11E-07 5.61E-07]	1.99E-07	[-7.35E-09 4.05E-07]
w	0.2732	[0.2428 0.3035]	0.2732	[0.2422 0.3042]

Appendix C

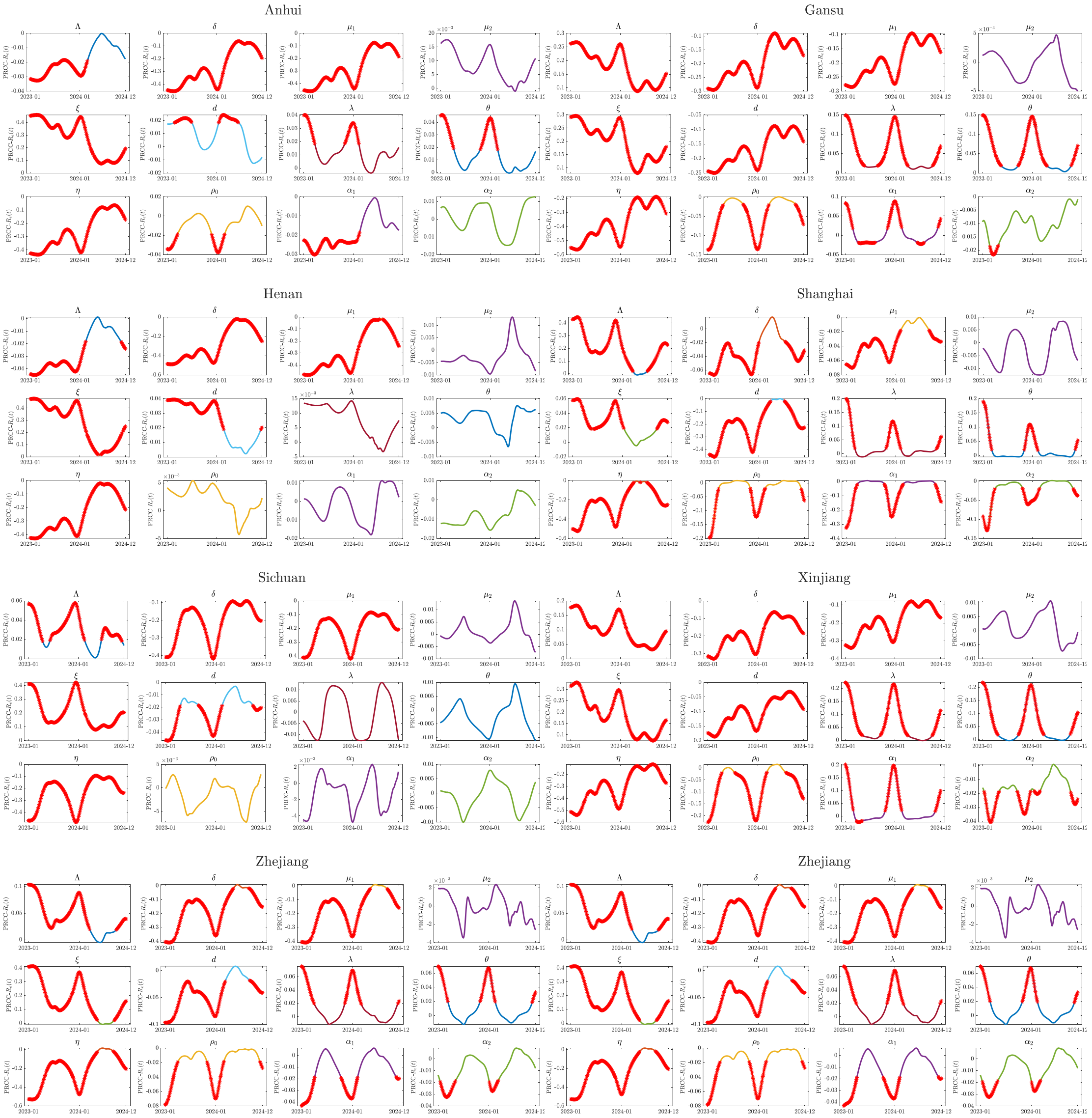

Fig. C1 Temporal significance of PRCC between model parameters and the effective reproduction number $R_e(t)$ across different provinces. Red stars on the curve denote time points with statistically significant associations with the parameter ($p < 0.01$).

Appendix D

Fig A2, Fig D1 and Fig D2 report the correlation analyses between the Baidu Search Index (BSI) and three model-related variables across eight provinces in China: (i) observed influenza cases, (ii) MCMC-estimated recovered individuals, and (iii) MCMC-estimated susceptible individuals. These analyses quantify the strength and statistical significance of the associations between BSI and each epidemic state variable.

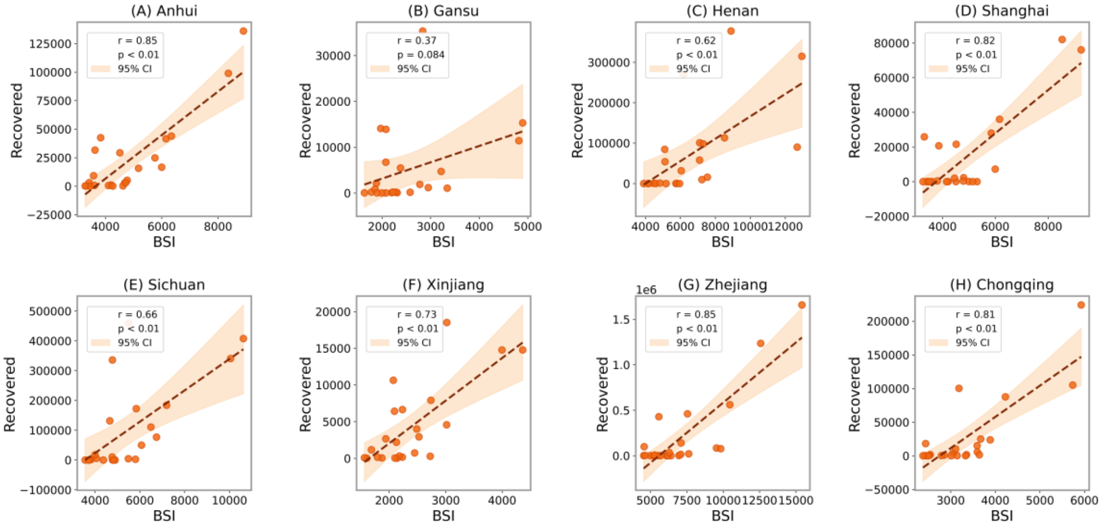

Fig. D1 Correlation analysis between recovered cases and BSI across different provinces of China (2023–2024).

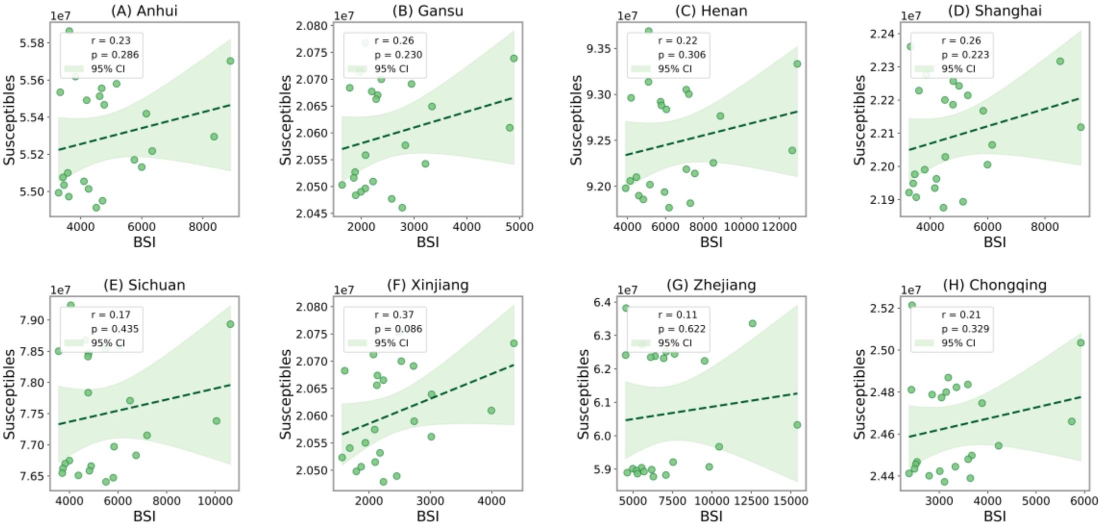

Fig. D2 Correlation analysis between susceptibles and BSI across different provinces of China (2023–2024).

Appendix E

To further contextualize the regional differences in media–response effects, we compiled several representative indicators from the National Bureau of Statistics of China for both Xinjiang and Zhejiang[1]. These indicators include internet broadband access ports, broadband user counts, mobile internet users, and high school student population. Together, they reflect each region's information infrastructure, media accessibility, and general educational environment.

Fig E1 presents a visual comparison between Zhejiang and Xinjiang for the four indicators. Subplots (A) and (B) show that Zhejiang has substantially higher broadband access capacity and user numbers, indicating wider and faster information dissemination channels. Subplot (C) reveals that Zhejiang's mobile internet population is more than three times that of Xinjiang throughout the observation period, suggesting much stronger exposure to online media. Subplot (D) shows that Zhejiang also

[1] National Bureau of Statistics of China (NBS). 2025 [cited 2025 Nov 18]. https://data.stats.gov.cn/

maintains a larger and more stable high school student population, which may reflect higher overall educational levels and greater capacity to interpret and respond to public–health information.

Taken together, these indicators suggest that Xinjiang's weaker response to media attenuation may stem from its relatively lower digital infrastructure, more limited internet access, and comparatively lower educational levels, all of which may reduce the effectiveness of media-based risk communication.

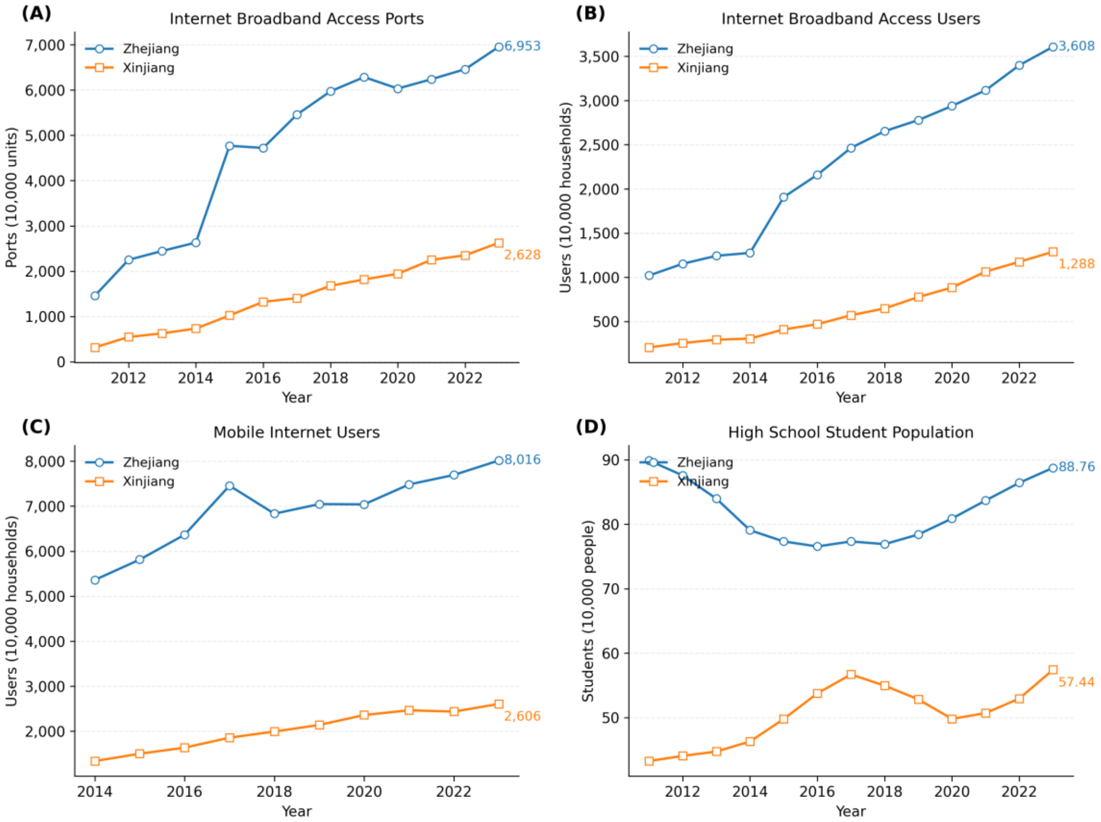

Fig. E1 Comparison of Information Infrastructure and Educational Indicators between Xinjiang and Zhejiang (2011–2023).

To further examine the structural determinants underlying regional heterogeneity, we also conducted an exploratory correlation analysis between the province-specific estimates of δ and μ_1 and several socioeconomic indicators across eight provinces in China. Specifically, we considered four representative indicators: Broadband Ports Penetration Rate, Mobile Internet Penetration Rate, Log(GDP per capita), and Broadband Penetration Rate. These variables capture different aspects of digital infrastructure, and economic development, and were summarized at the provincial level using representative values for 2024, with internet-related variables standardized by resident population to improve comparability.

Figures E2 and E3 summarize the results of this analysis. Figure E2 contains four subplots describing the relationships between δ and the four socioeconomic indicators, while Figure E3 presents the corresponding relationships for μ_1. The results show that μ_1 exhibits generally positive associations with economic development and digital infrastructure, indicating that provinces with higher GDP and better internet accessibility tend to show stronger public information-search behavior. In contrast, δ does not display stable or significant correlations with any single socioeconomic indicator, suggesting that regional differences in media responsiveness are unlikely to

be determined by one factor alone. Rather, they likely reflect the combined influence of multiple structural determinants, including public risk perception, behavioral response patterns, regional information environments, and broader social conditions.

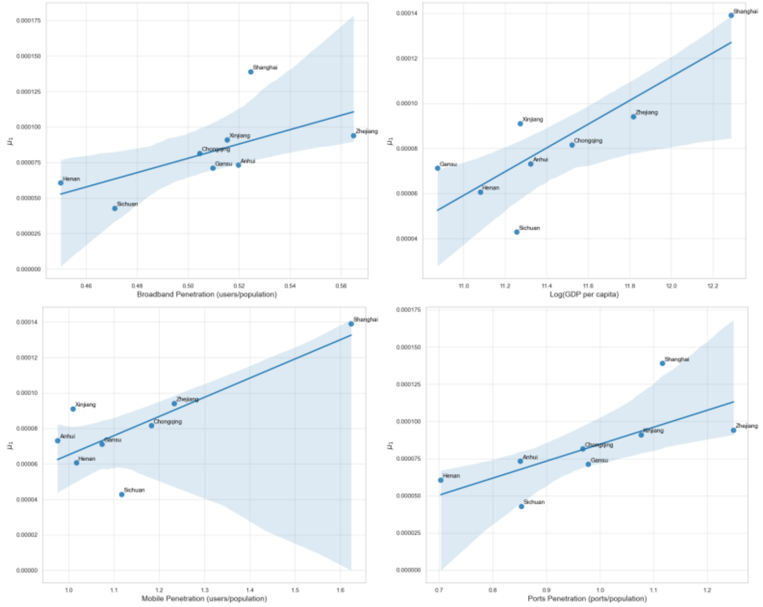

Fig E2. Relationships between the estimated parameter μ_1, representing the contribution of susceptible individuals to search behavior, and five provincial socioeconomic indicators across eight provinces in China (2024). The solid line represents the fitted linear regression, and the shaded area indicates the 95% confidence interval. (a) Broadband Penetration Rate (users/population). (b) Broadband Ports Penetration Rate (ports/population). (c) Mobile Internet Penetration Rate (users/population). (d) Log(GDP per capita). Each point corresponds to a province and is labeled accordingly.

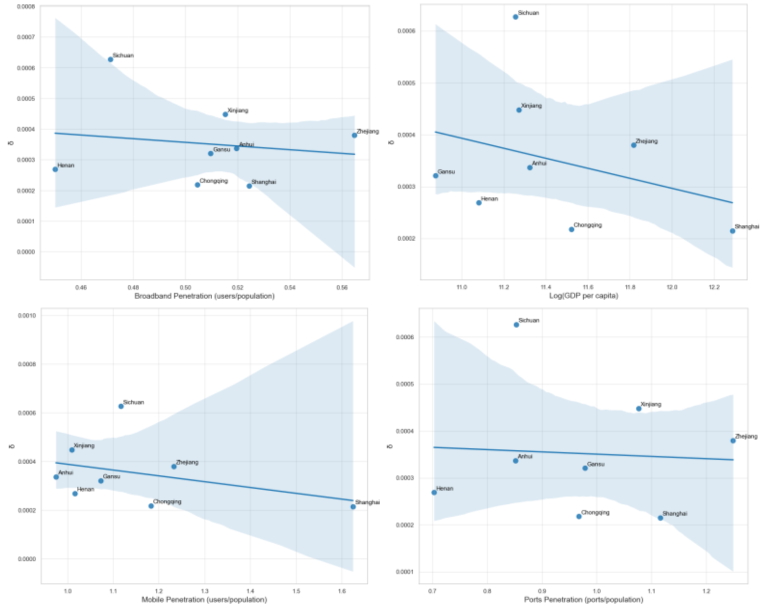

Fig E3. Relationships between the estimated media response parameter δ and five provincial socioeconomic indicators across eight provinces in China (2024). The solid line represents the fitted linear regression, and the shaded area indicates the 95% confidence interval. (a) Broadband Penetration Rate (users/population). (b) Broadband Ports Penetration Rate (ports/population). (c) Mobile Internet Penetration Rate (users/population). (d) Log(GDP per capita). Each point represents a province and is labeled accordingly.

Overall, these findings suggest that μ_1 more directly reflects the influence of

provincial economic development and information infrastructure on public search behavior, whereas δ appears to be shaped by a more complex combination of structural factors. The combined evidence from the Xinjiang–Zhejiang comparison and the province-level correlation analysis provides a more comprehensive explanation for the observed regional heterogeneity in media responsiveness.

Appendix F

This section addresses the interaction effect between temperature and precipitation. As shown in Fig F1, a significant positive correlation between temperature and precipitation is observed in most provinces, which aligns with the expectation that higher temperatures are often accompanied by increased precipitation.

To better capture the effects of this interaction, four different MAT (mean annual temperature) and MTP (mean total precipitation) interaction scenarios were designed: (i) MAT+3°C and 1.3×MTP; (ii) MAT+1.5°C and 1.15×MTP; (iii) MAT-1.5°C and 0.85×MTP; (iv) MAT-3°C and 0.7×MTP.

These scenarios allow for a comparison of the effects of the interaction with those of independent MAT or MTP disturbances under the same perturbations, providing insights into how the interaction influences the results.

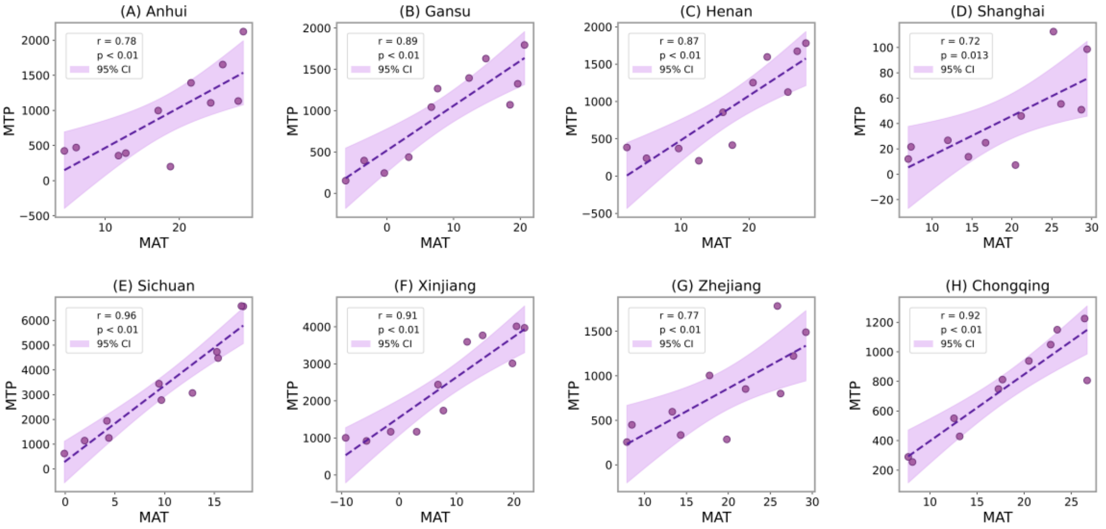

Fig. F1 Correlation analysis between monthly average temperature (MAT) and monthly total precipitation (MTP) across different provinces of China (2023).

Fig 5 presents the relative changes in influenza cases across provinces under the four scenarios, with Case 1 to Case 4 representing different disturbance intensities. In the figure, the pink bars represent the MAT and MTP interaction scenario, the green bars represent the MAT-only disturbance, and the blue bars represent the MTP-only disturbance. The figure clearly shows significant differences in influenza case changes across provinces under the different scenarios. Specifically, when considering the interaction between temperature and precipitation, the changes in influenza cases are more pronounced compared to the individual MAT or MTP disturbances. This result reinforces the idea that cold and dry conditions can significantly promote influenza transmission, and this effect is amplified when temperature and precipitation interact.

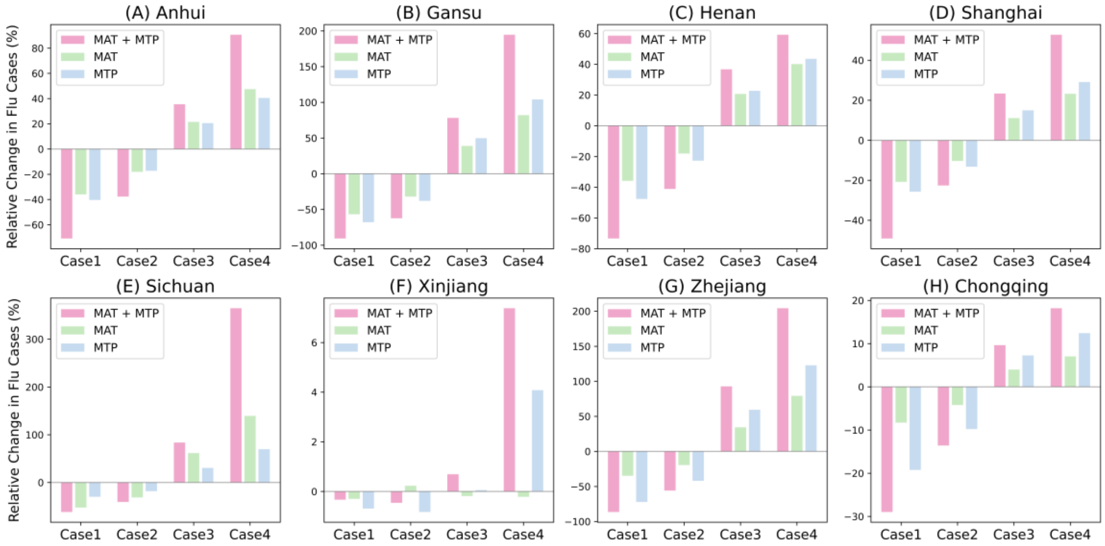

Fig. F2 Relative changes in influenza cases across provinces under different MAT and MTP interaction scenarios. Case 1: MAT+3°C and 1.3×MTP; Case 2: MAT+1.5°C and 1.15×MTP; Case 3: MAT-1.5°C and 0.85×MTP; Case 4: MAT-3°C and 0.7×MTP. The pink bars represent the interaction between MAT and MTP, the green bars represent the MAT-only disturbance, and the blue bars represent the MTP-only disturbance.
